# Helical coiling of metaphase chromatids

**DOI:** 10.1093/nar/gkad028

**Published:** 2023-03-02

**Authors:** Ivona Kubalová, Amanda Souza Câmara, Petr Cápal, Tomáš Beseda, Jean-Marie Rouillard, Gina Marie Krause, Kateřina Holušová, Helena Toegelová, Axel Himmelbach, Nils Stein, Andreas Houben, Jaroslav Doležel, Martin Mascher, Hana Šimková, Veit Schubert

**Affiliations:** Leibniz Institute of Plant Genetics and Crop Plant Research (IPK), Gatersleben, D-06466 Seeland, Germany; Leibniz Institute of Plant Genetics and Crop Plant Research (IPK), Gatersleben, D-06466 Seeland, Germany; Institute of Experimental Botany of the Czech Academy of Sciences, Centre of the Region Haná for Biotechnological and Agricultural Research, Olomouc 77900, Czech Republic; Institute of Experimental Botany of the Czech Academy of Sciences, Centre of the Region Haná for Biotechnological and Agricultural Research, Olomouc 77900, Czech Republic; Daicel Arbor Biosciences, Ann Arbor, MI, USA; Chemical Engineering Department, University of Michigan, Ann Arbor, MI, USA; Leibniz Institute of Plant Genetics and Crop Plant Research (IPK), Gatersleben, D-06466 Seeland, Germany; Institute of Experimental Botany of the Czech Academy of Sciences, Centre of the Region Haná for Biotechnological and Agricultural Research, Olomouc 77900, Czech Republic; Institute of Experimental Botany of the Czech Academy of Sciences, Centre of the Region Haná for Biotechnological and Agricultural Research, Olomouc 77900, Czech Republic; Leibniz Institute of Plant Genetics and Crop Plant Research (IPK), Gatersleben, D-06466 Seeland, Germany; Leibniz Institute of Plant Genetics and Crop Plant Research (IPK), Gatersleben, D-06466 Seeland, Germany; Center of Integrated Breeding Research (CiBreed), Department of Crop Sciences, Georg-August-University, D-37075 Göttingen, Germany; Leibniz Institute of Plant Genetics and Crop Plant Research (IPK), Gatersleben, D-06466 Seeland, Germany; Institute of Experimental Botany of the Czech Academy of Sciences, Centre of the Region Haná for Biotechnological and Agricultural Research, Olomouc 77900, Czech Republic; Leibniz Institute of Plant Genetics and Crop Plant Research (IPK), Gatersleben, D-06466 Seeland, Germany; German Centre for Integrative Biodiversity Research (iDiv), Halle-Jena-Leipzig, D-04103Leipzig, Germany; Institute of Experimental Botany of the Czech Academy of Sciences, Centre of the Region Haná for Biotechnological and Agricultural Research, Olomouc 77900, Czech Republic; Leibniz Institute of Plant Genetics and Crop Plant Research (IPK), Gatersleben, D-06466 Seeland, Germany

## Abstract

Chromatids of mitotic chromosomes were suggested to coil into a helix in early cytological studies and this assumption was recently supported by chromosome conformation capture (3C) sequencing. Still, direct differential visualization of a condensed chromatin fibre confirming the helical model was lacking. Here, we combined Hi-C analysis of purified metaphase chromosomes, biopolymer modelling and spatial structured illumination microscopy of large fluorescently labeled chromosome segments to reveal the chromonema - a helically-wound, 400 nm thick chromatin thread forming barley mitotic chromatids. Chromatin from adjacent turns of the helix intermingles due to the stochastic positioning of chromatin loops inside the chromonema. Helical turn size varies along chromosome length, correlating with chromatin density. Constraints on the observable dimensions of sister chromatid exchanges further supports the helical chromonema model.

## INTRODUCTION

Chromosomes occupy distinct territories within interphase nuclei, with little constraints on their shape. Cell division entails the condensation of interphase chromosomes into rod-like structures, compacting chromatin up to 1000-fold. The regularity and ubiquity of this process among eukaryotes likely mean that it is governed by general laws. How many different molecular mechanisms act together to establish the distinct form and physical properties of mitotic chromosomes is currently under intensive investigation (1).

Several models have been proposed to describe the higher-order structure of metaphase chromosomes based on data obtained using a range of molecular and microscopy methods (2). These models are categorized as helical and non-helical. Helical models assume that the chromatin in each sister chromatid at metaphase is arranged in a helix (3–10), whereas non-helical models suggest that chromatin is folded within the chromatids without forming a helix (11–17).

The helical structure of metaphase chromosomes was first observed in the plant *Tradescantia virginica* L. by Baranetzky (18). The helically coiled chromatin thread that he described was named chromonema (from chromo=color + Greek nēma=thread; plural: chromonemata) (19). Using light microscopy, the chromonema continued to be reported, over several decades, in treated and native chromosomes of diverse plant species and cultured human leukocytes (3,4,20–24). But later microscopy observations could not detect the chromonema coiling in Drosophila (25), or other mammalian cell lines (16,26). Most recently, genome-wide chromatin contact profiles, gleaned from chromosome conformation capture sequencing (Hi-C), have rekindled interest in helical models. They indicated a helical organization for chromosomes of chicken (10), axolotl (8) and HeLa cells (10,17), suggesting that this arrangement might be a common feature among vertebrates. Using computational polymer models that reproduce the stochastic pattern of quantified Hi-C chromatin contacts, Gibcus et al. described further details of the organization of late-prometaphase chromosomes of chicken – likely comprised of 12 Mb long helical turns with a 200 nm pitch (10).

Besides Hi-C, advanced microscopy and cytological techniques can inform about the higher-order spatial (3D) chromatin organization of mitotic chromosomes. Fluorescence *in situ* hybridization (FISH) using oligonucleotide probes (oligo-FISH) is an efficient method for examining whole chromosomes and their specific regions (27–29). The application of super-resolution microscopy techniques in combination with oligo-FISH led to the characterization of sub-chromosomal structures (30). The arrangement of chromatin in sister chromatids in spontaneously occurring sister chromatid exchanges (SCEs) can be studied via the differential incorporation of DNA base analogues, such as 5-ethynyl-2′-deoxyuridine (EdU) (31), into chromosomes during replication (32–34). This method differentially labels sister chromatids (harlequin staining) and can be combined with FISH (35,36).

To study the higher-order structure of plant mitotic chromosomes, we used the large chromosomes of barley (*Hordeum vulgare* L.; 2*n* = 14; 1C = 4.88 Gb) (37) as a model. Our analysis using a combination of metaphase chromosome-derived Hi-C data, oligo-FISH, SCEs detection, super-resolution microscopy and polymer simulation revealed that sister chromatids are composed of chromatin helices of identical handedness. The helical turns cover 20–38 Mb, creating a ∼400 nm thick fibre, which we identify as the chromonema.

## MATERIALS AND METHODS

### Plant material

Barley (*Hordeum vulgare* L.) cv. Morex seeds were obtained from the Gene Bank of the Leibniz Institute of Plant Genetics and Crop Plant Research Gatersleben, Germany.

### Preparation and sequencing of chromosome Hi-C libraries

Suspensions of barley metaphase chromosomes were prepared from root tip meristems after cell cycle synchronization as described in (38) with the following modifications. Barley seeds were germinated for 2 days at 4°C, followed by incubation at 25°C for 3 days. The roots were fixed in 2% formaldehyde in 1× PBS buffer for 12 min at 5°C. Fixed metaphase chromosomes were released by mechanical homogenization into LB01 buffer (37) and stained by 4',6-diamidino-2-phenylindole (DAPI) at a final concentration of 2 μg/ml. The chromosomes were purified by sorting using a FACSAria SORP flow cytometer (Becton Dickinson, San Jose, CA USA). The initial gating was performed on FSC-A versus DAPI-A parameters. To discriminate doublets, the chromosome gate was drawn on a DAPI-A versus DAPI-W scatterplot (Supplementary Figure S1).

Three replicates of chromosome Hi-C libraries were prepared. For each replicate, five million chromosomes were flow sorted into a 15-ml Falcon tube with 2 ml LB01, centrifuged at 500 *g* for 30 min at 4°C and the supernatant was removed except for 20 μl. The pelleted chromosomes were gently resuspended in the remaining supernatant, diluted with 8 ml ddH_2_O and spun down at 500 *g* for 30 min at 4°C. The supernatant was removed except for 20 μl. The pelleted chromosomes were gently resuspended, and the entire sample was transferred to a 0.2 ml Eppendorf tube. Further steps were carried out with an Arima Hi-C kit (Arima Genomics, San Diego, CA, USA) according to the manufacturer's protocol. The sequencing libraries were prepared with a NEBNext Ultra II DNA library preparation kit (NEB, Ipswitch, MA USA) with 10 cycles of PCR amplification. Libraries were sequenced on an Illumina NovaSeq 6000 instrument (Illumina, San Diego, CA, USA) in 150-bp paired end mode. The parameters of the generated Hi-C data are summarized in Supplementary Table S1.

The data for chromosome model simulation were generated by an alternative protocol. Eight million chromosomes were sorted into a 15-ml Falcon tube with 2 ml isolation buffer (39), centrifuged at 500 *g* for 30 min at 4°C and the supernatant was removed except for 45 μl. Pelleted chromosomes were gently resuspended in the remaining supernatant and mixed with low-gelling agarose to create a 90-μl 1% agarose plug. The plug was washed twice for 30 min in 2 ml TE buffer (10 mM Tris–HCl, 1 mM EDTA, pH 8) on ice. Subsequent Hi-C library preparation steps, including DNA digestion, biotin fill-in, ligation and chromatin release from the plug, were performed according to the ‘In situ Hi-C in agar plugs protocol’ (40) with minor modifications: the DNA was digested by 800 U HindIII, and biotin-14-dATP was replaced by biotin-14-dCTP. The completed Hi-C library was sequenced on the HiSeq2500 system in the 100-bp paired-end mode.

Hi-C reads were mapped against the barley cv. Morex genome version 2 (v2; (41)) using a previously described computational pipeline (41,42). Contact matrices were visualized in the R statistical environment (43).

### Contact probabilities from Hi-C data

All contacting pairs from Hi-C data were counted and mapped to the Morex genome version 2 (v2), as a function of the genomic distance between them (*d*). All values of distance *d* were split into bins on a logarithmic scale from 100 kb to 1 Gb. The contact probability of each bin was calculated as the sum of all observed pairs within the distance range of this bin, divided by the number of all genomic loci within the same distance range.

### Quantification of Hi-C local contacts in metaphase chromosomes

**Figure 1.**
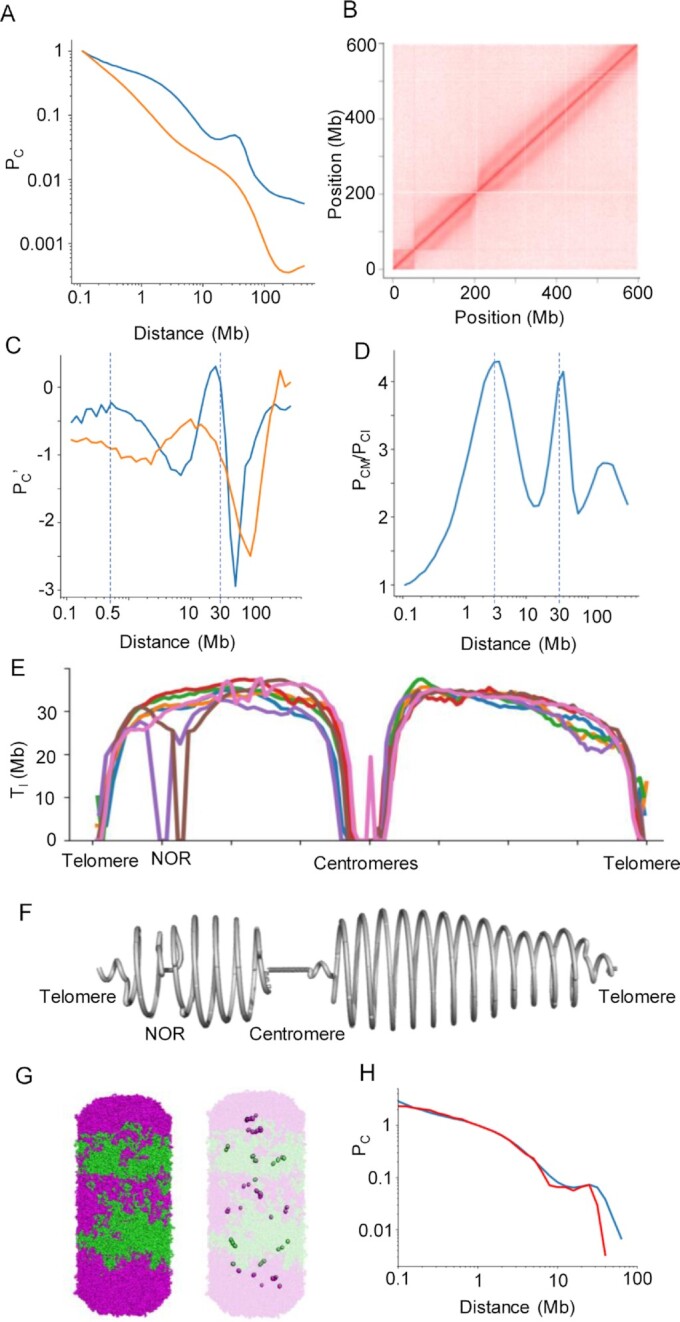
Hi-C data analysis and polymer model of barley metaphase chromosomes. (**A**) Hi-C contact probability (P_C_) determined for barley chromosome 5H at metaphase (blue) and interphase (orange). The statistics of all Hi-C datasets are shown in Supplementary Table S1. (**B**) Hi-C contact matrix of metaphase chromosome 5H. (**C**) Derivative of the log-transformed contact probability in metaphase (blue) and interphase (orange). The two dashed lines mark: 500 kb, where the derivative is close to null and the probability decay brakes, and 30 Mb, where the derivative is null, and the probability reaches a peak. (**D**) Contact probability ratio between metaphase (*P*_CM_) and interphase (*P*_CI_). The dashed line marks high contact peaks that distinguish metaphase from interphase. (**E**) Turn length (T_l_) along all chromosomes based on the local Hi-C contacts (Supplementary Table S2). The chromosome positions were scaled to a relative distance from the centromere. (**F**) A helical model illustrating the variation in turn length for chromosome 5H. (**G**) Bottle-brush polymer model (equilibrated after 100 000 steps) representing barley metaphase chromosomes. Five turns with intercalating magenta and green colours are shown on the left and the bases of the major loops are highlighted on the right. (**H**) Contact probability comparing Hi-C data (blue) and the polymer model (red).

The chromosomes were divided into 5-Mb-long non-overlapping regions. For each region, we considered only Hi-C pairs with at least one side of the contacting pair inside of this region or with both sides of this pair spanning this region (Supplementary Figure S2). For each region, the pairs were counted based on the genomic distance between them (*d*). The counted pairs as a function of *d* are well described by the sum of an exponential and a Gaussian distribution. The Gaussian distribution coincides with the bump characteristic of a helical arrangement (Figure 1A), and its centre indicates the turn length of the helical arrangement around the analysed region. The centre of the Gaussian distribution was assigned as the turn length for each region (Supplementary Figure S3) and was used to build the graphics in Figure 1 and Supplementary Figure S4.

### Building the helical path with a changing radius and estimating the number of turns

The monomers selected as the loop basis were constrained to a helical path in the Cartesian space. A turn length (in base pairs) *I_i_* was assigned to each monomer i according to the values calculated from the Hi-C data. With this value, we calculated how many monomers (*n_i_*) from the helical path would fit in this turn, with monomer size (in base pairs) *m* and the average major loop size (in monomers) *I_o_* (equation 1). Each monomer was then assigned a height *z_i_* and an angle *θ_i_* relative to the previous monomer. The height *z_i_* is equal to the constant turn height (400 nm) divided by *n_i_*_,_ and the angle *θ_i_* is the full turn (360°) divided by *n_i_* (equation 2). The radius *r_i_* was calculated from the turn length (*n_i_* multiplied by the distance *d* between monomers) and the turn height as in equation (3), from the Pythagorean theorem. The cartesian coordinates were then retrieved by these cylindrical coordinates.


(1) $$\begin{equation*}{n}_i = \ {l}_i/m/{l}_0\end{equation*}$$

(2) $$\begin{equation*}{z}_i = \ {z}_{i - 1} + 400/{n}_i, \quad\quad {\theta }_i = \ {\theta }_{i - 1} + 360/{n}_i\end{equation*}$$

(3) $$\begin{equation*}{\left( {d{n}_i} \right)}^2 = \ {\left( {2\pi {r}_i} \right)}^2 + {400}^2\end{equation*}$$

The number of turns is the number of cycles in the function cos(*θ*), using 1 Mb as the monomer size and 1 monomer as the average major loop size.

### Plant growth and treatment for FISH

Barley seeds were incubated for three days in the dark at room temperature. Roots were collected in cold tap water and incubated on ice for 24 h. The roots were transferred into ethanol:acetic acid (3:1) fixation solution and placed under a vacuum for 5 min. The roots were stored in this solution overnight at room temperature (RT).

### Plant growth and treatment for EdU labeling of SCEs

Barley seeds were incubated for three days in the dark, followed by incubation in 20 μM EdU in Hoagland medium for 17 h at RT. The seedlings were transferred into fresh Hoagland medium and incubated for 19 h in the dark at RT (recovery phase). Roots were cut from the seedlings and incubated in ice-cold water for an additional 24 h. Finally, the roots were fixed in ethanol:acetic acid (3:1) for 5 min under vacuum and overnight at RT.

### Chromosome spread preparation

Selected roots were washed twice in ice water for 5 min and once in a citric buffer for 5 min. The roots were digested in an enzyme mixture [1% pectolyase (Sigma); 1% cytohelicase (Sigma); 0.7% cellulase R-10 (Duchefa) and 0.7% cellulase (Calbiochem) in 0.1 M citric buffer] at 37°C for 45 min. Following enzymatic digestion, the roots were washed twice in ice-cold water for 5 min and twice in ice-cold ethanol for 5 min. Root tips without root caps were collected into the tube containing ethanol:acetic acid (1:3) and mashed. 7 μl of this suspension was dropped onto a cold wet slide, transferred to a hot plate (55°C) and fixed with 25 μl of ethanol:acetic acid (3:1). The slides were allowed to dry at least for 1 h at RT.

To preserve the native volume of the chromosomes, metaphase chromosomes were flow-sorted into 1× meiocyte buffer A (1× buffer A salts, 0.32 M sorbitol, 1× DTT, 1× polyamines) and subsequently embedded into a 5% polyacrylamide gel as described (44,45) with minor modifications (46).

### FISH

To detect half and full-helix turns within chromosome arm 5HL by fluorescence microscopy, oligo-FISH probes were designed against the chromosome-scale sequence assembly of the barley cv. Morex genome assembly version 1 (Morex v1; (42,47)) using Daicel Arbor Biosciences’ proprietary software. Briefly, target sequences were fragmented into 43–47 nucleotide-long overlapping probe candidate sequences that were compared to the rest of the genome sequence to exclude any candidates with potential cross-hybridization based on a predicted *Tm* of hybridization. Non-overlapping target-specific oligonucleotides were selected for the final probe sets and synthesized as myTAGs® Labeled Libraries (Daicel Arbor Bioscience, Ann Arbor, MI, USA).

**Figure 2.**
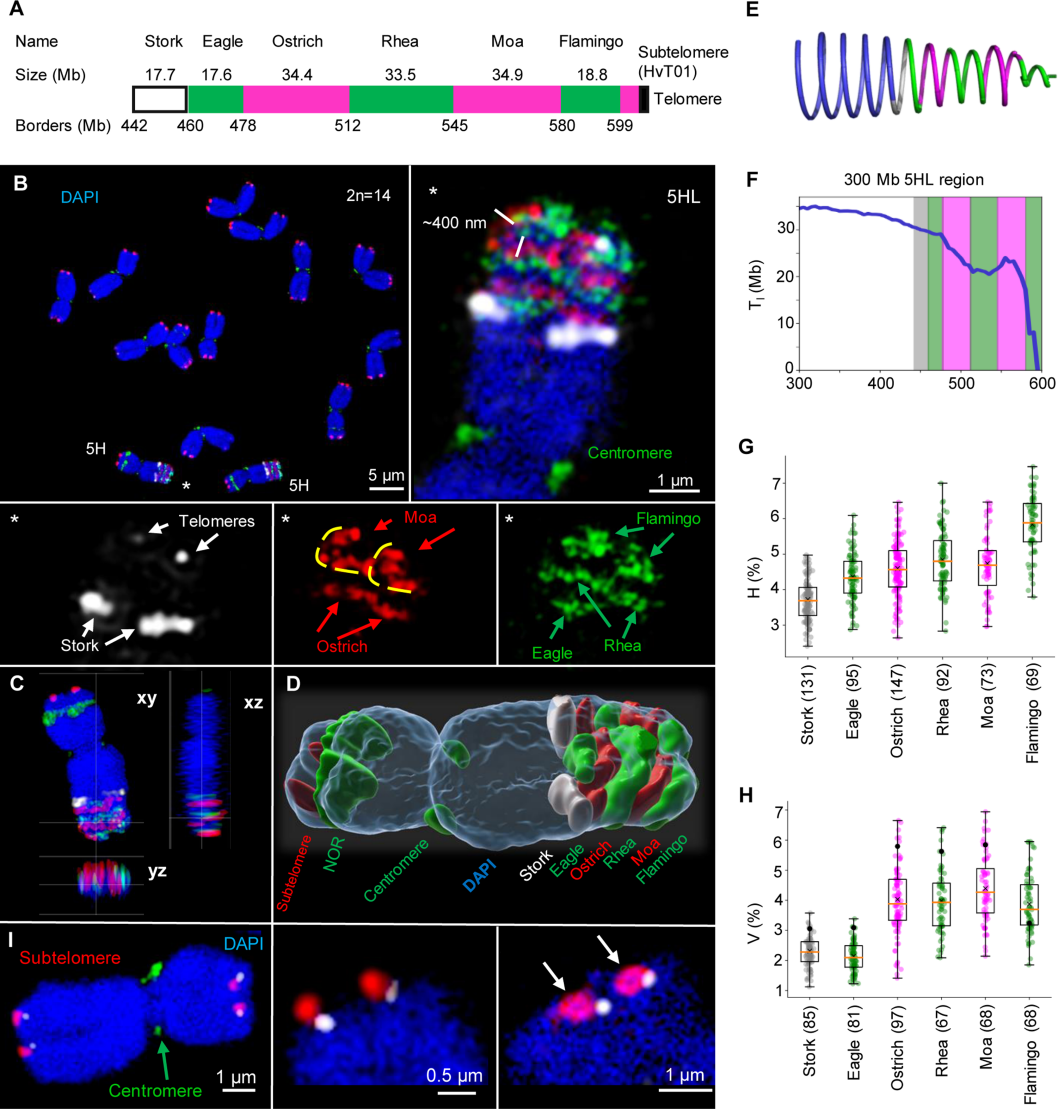
FISH confirms the helical organization of barley metaphase chromosomes. (**A**) Design of the oligo-FISH probes covering the 157 Mb-long region of chromosome 5HL (Supplementary Table S3). (**B**) FISH-labeled metaphase chromosomes. The enlarged oligo-painted region of 5HL (asterisks) shows the chromatin arrangement in both sister chromatids as predicted by the Hi-C-based helical chromatin arrangement model (Figure 1G). Due to chromosome tilting, Moa signals show, in a top-side view, a turn of the ∼400 nm thick chromatin fibre (marked with yellow lines). (**C**) Ortho-view (Supplementary Movie S3) and (**D**) surface rendering (Supplementary Movie S5) of the same 5H homologue. (**E**) Helical arrangement of the target region illustrating the changes of the turn lengths (T_l_). (**F**) T_I_ calculated from the Hi-C data, across a 300 Mb region of 5HL encompassing the designed oligo-FISH probes. The regions covered by each probe are coloured according to the probe colour. (**G**) Relative heights (H) of the measured oligo-FISH probe signals as a percentage of the whole chromosome height. (**H**) Relative volume (*V*) of the measured oligo-FISH probe signals as a percentage of the whole chromosome volume. The black dots are the percentage of the DNA content of the probes relative to the entire chromosome. (**I**) Positions of telomeres (white) and subtelomeres (red) vary at both termini of different chromosomes. Subtelomeres form ring-like structures (right, arrows). Chromatin was counterstained with DAPI (blue). For (G) and (H), the total number of measured chromosomes per oligo probe are in parentheses.

To validate the lengths of intervals covered by the designed oligo-FISH probes, we verified the completeness of the Morex v2 genome assembly of the analysed region of 5H using an optical map constructed from barley cv. Morex on the Saphyr platform of Bionano Genomics (Supplementary Figure S5). The optical map contigs were aligned to the selected interval (442–599 Mb) of the chromosome 5H pseudomolecule using RefAligner version 9232 (Bionano Genomics). Query-to-anchor comparison was performed with default parameters and a *P*-value threshold of 1e−10. The alignment was visualized using the Bionano Access version 1.5. The coordinates from the original design were replaced and are now based on the improved Morex assembly v2 (41). The labeled region starts at position 442.1 Mb. For simplicity, the designed probes were given the names of different bird species and labeled in different colours: Stork-Atto647N, Eagle-Alexa488, Ostrich-Atto594, Rhea-Alexa488, Moa-Atto594 and Flamingo-Alexa488 (Figure 2A, Supplementary Figure S6; Supplementary Table S2).

Subtelomeres were labeled with the HvT01 oligo-probe (48,49) using TexasRed, and telomeres were labeled with the *Arabidopsis* telomere-type oligo probe (50) using Cy5. The centromeres were labeled with the probe for centromeric repeats (GA)_15_ (51) using FAM, and the NORs were Alexa488-labeled with the 45S rDNA probe using the clone pTa71 (52).

For FISH, first, the slides were incubated in 45% acetic acid at RT for 10 min and washed in 2× SSC at RT for 10 min. When strong background signals due to cytoplasm were present, 50 μl of pepsin solution (0.1% in 0.01 M HCl) was added to the slide, which was then covered with parafilm and incubated in a wet chamber at 37°C for 10 min. The slides were washed twice in 2× SSC for 5 min and post-fixed with 4% formaldehyde in 2× SSC at RT for 10 min. After, the slides were then washed 3 times in 2× SSC at RT for 4 min and immediately dehydrated in an ethanol gradient (70%, 85% and 100%, 2 min each). Then, the slides were air-dried for at least 1 h. In the meantime, all selected oligo probes were pooled into a microtube, and the solutions were evaporated using a SpeedVac concentrator (Eppendorf). Per slide, the probes were reconstituted in 1 μl of ddH_2_O and 19 μl of hybridization mixture (50% formamide; 10% dextran sulfate; 10% salmon sperm DNA; 2× SSC). The entire volume of the reconstituted probe was added to the slide, covered with a coverslip and sealed with rubber cement. The slides were incubated for 20 min at 37°C and denatured on a hot plate (70°C) for 3 min. Finally, the slides were directly placed into a wet chamber and hybridized for 22 h at 37°C. Post-hybridization washing was carried out as follows: the slides were briefly washed in 2× SSC at RT to remove the coverslip, washed with shaking at 58°C for 20 min, followed by 5 min in 2× SSC at RT, dehydrated in an ethanol series (70%, 90% and 96%, 2 min each), air-dried in the dark and counterstained with 8 μl DAPI (2 μg/ml in Vectashield).

### Combination of EdU labeling and FISH

Seedling growth and treatment were performed as described for the EdU labeling experiment. Slides containing metaphase spreads with EdU-labeled SCEs were pre-selected and used for oligo-FISH. Depending on the cytoplasm background, the slides were incubated in 45% acetic acid at RT for 10 min and washed in 2× SSC at RT for 10 min or directly post-fixed with 4% formaldehyde in 2× SSC at RT for 10 min. The subsequent steps were identical to the FISH procedure described above.

### SMC2A antibody preparation and specificity proof

Condensin SMC2A antibodies (SMC2Arb97) were raised by the LifeTein company (USA) in rabbits against the peptide KSKRDEATAA EKELKARTKD-C. SMC2A is encoded by the barley gene *HORVU.MOREX.r3.3HG0304800*. Polyclonal antibodies were diluted in 1× PBS with 0.02% sodium azide to obtain a stock solution of 5 mg/ml.

The antibody specificity was shown via peptide competition on flow-sorted 4C nuclei isolated from roots. The synthetic peptide used for the rabbit immunization was reconstituted in 1× PBS with 0.02% sodium azide to obtain a stock solution of 5 mg/ml. The antibodies were mixed with the peptide in antibody solution (1% BSA, 0.01% Triton X-100, 1× PBS) to the final dilution of 1:100. The peptide concentration used was 1:100 and 1:50. The mixture was incubated overnight at 4°C. Next day, the slides with chromosomes and flow-sorted 4C nuclei were blocked (5% BSA, 0.03% Triton X-100, 1× PBS for 1.5 h at room temperature) and incubated with the antibody/peptide mixture overnight at 4°C. Afterwards, the slides were three times washed in 1× PBS and incubated with secondary donkey anti-rabbit Alexa488 antibodies (1:200, #711–545-152 Jackson ImmunoResearch), diluted in antibody solution, for 1 h at 37°C. Next, the slides were washed three times in 1× PBS at RT followed by dehydration in an ethanol gradient (70%, 85% and 100%), each step 1 min. Air-dried slides were counterstained with DAPI and subjected to microscopy.

### Super-resolution microscopy and measurement of FISH signal volumes

To detect the ultrastructural chromatin organization of chromosomes at a resolution of ∼120 nm (super-resolution achieved with a 488 nm laser excitation), spatial structured illumination microscopy (3D-SIM) was performed with an Elyra PS.1 microscope system with a 63×/1.4 Oil Plan-Apochromat objective using the ZENBlack software (Carl Zeiss GmbH). Images were captured separately for each fluorochrome using the 642, 561, 488 and 405 nm laser lines for excitation and appropriate emission filters (53). Maximum intensity projections of whole cells were calculated using the ZENBlack software. Zoomed-in sections were presented as single slices to indicate the subnuclear chromatin structures at super-resolution. 3D rendering to produce spatial animations was performed based on SIM image stacks using the Imaris 9.6 (Bitplane) software. The FISH signal and DAPI-labeled whole chromosome volumes were generated and measured with the Imaris tool ‘Surface’.

### Polymer simulation

In all simulations, the chromatin was modelled as a beads-on-a-string homo-polymer, where each bead corresponds to one nucleosome plus linker (200 bp). Molecular Dynamics simulations (MD) were performed using the OpenMM Python application programming interface (54) and the OpenMM-lib library (https://github.com/mirnylab/). The motion of the polymer was simulated based on Langevin dynamics, with a temperature of 300 K, a collision frequency of 0.001 ps^−1^ and a variable time step (10). In the simulation, three internal forces were applied to the polymer, following the parameters used by Gibcus *et al.* (10): (i) a harmonic force covalently binding two neighbouring beads separated by 10 nm average distance (consistent with a 10 nm chromatin fibre) and 1 nm wiggle distance; (ii) a harmonic angular force between three sequential beads with a spring constant of 1 k_B_T/rad^2^ and (iii) a polynomial repulsive force allowing for the crossing of the fibre by setting an energy truncation value of 1.5 k_B_T, when the distance between two non-bonded beads is zero. In the bottle-brush model, the entire polymer is organized into side-by-side major loops divided into side-by-side minor loops, as proposed by Gibcus *et al.* (10). The lengths of both types of loops were randomly chosen, around a pre-determined average, following an exponential distribution. Two external forces were applied to constrain the polymer in a cylindrical bottle-brush model: (i) the monomers forming the base of the major loops were tethered to a helical path by a harmonic potential with a spring constant of 4 k_B_T/nm^2^ and (ii) this helical path was at the centre of a cylinder, whose radius secured a volume of 11^3^ nm^3^ per monomer, and whose boundaries were defined by a harmonically increasing potential with a spring constant of 10 k_B_T/nm^2^. Apart from these characteristics, the bottle-brush model is defined by four parameters, which seem specific for each species: (i) the average length of major loops; (ii) the average length of minor loops; (iii) the turn height and (iv) the turn length of the helical path. To model barley chromosomes, we took these parameters from Hi-C data analysis and microscopy observations: 3 Mb, 500 kb, 400 nm and 30 Mb. All simulations started with a conformation where the bases of the major loops followed a calculated helical path with five turns, and the loops emerge radially. All monomers in the helical path were assigned cylindrical coordinates (angle, radius and height). These coordinates were calculated so that the monomers were equally separated given the specified total number of major loops and the turn height and then converted to Cartesian coordinates. The 10 nm distance between monomers in the helical path, as suggested by Gibcus *et al.* (10) did not fit with the specific parameters we found for the barley model, so we used a 50 nm separation. The simulation ran until the contact probability is equilibrated.

To better understand the influence of the separation of the monomers in the helical path we tested other values (20 and 100 nm). For these models we used cylindrical confinement with a 650 nm radius, closer to the observed in barley mitotic chromosomes in our microscopy experiments. This change was accompanied by a change in the energy truncation value of the repulsive force to 2.5 kbT. For each model, we ran three simulation replicates and present the average contact probability.

For the comparison between helical and half-helical arrangements we simulated a 10 Mb region with 2 Mb turn length, 100 nm turn height, 100 kb major loops and 10 kb minor loops (we chose these small values only for the sake of argument and to reduce computational cost). The radius of the helical path was set to 100 nm and no cylindrical confinement was set. The half-helical arrangement differed from the helical only in the position of the major loops’ basis—only the absolute values of the *x*-coordinate were considered in the conversion from cylindrical to Cartesian coordinates. Ten replicates of each model were used to calculate the contact probability.

### Contact probabilities of polymer models

To measure the contact probabilities of the equilibrated models, the final conformation of each simulation was calculated by counting all monomers spatially close to each other by no more than 51 nm, as in Gibcus *et al.* (10). These pairs of monomers were then grouped according to their distance in the linear genome. The sum of observed contacts in each group was divided by the sum of all possible contacts between two monomers separated by this group distance in the linear genome. The contact probability as a function of the linear genomic distance was directly compared to the experimental data obtained by Hi-C experiments upon normalization of all probability functions to be equal to 1 for 100 kb.

## RESULTS AND DISCUSSION

### Hi-C indicates that the mitotic chromatid is organized as a helix with variable turn lengths

We chose barley (*Hordeum vulgare* L.) for our work because it has large chromosomes (∼10 μm), and its mitotic chromosomes can be sorted in large numbers by flow-cytometry (Supplementary Figure S1). The distance-dependent decay of contact probability (*P*_C_) observed in the Hi-C data of purified barley metaphase chromosomes showed the same disruptions as in chicken macrochromosomes (Figure 1A, Supplementary Figure S7) (10). The visual correspondent of this pattern was the second diagonal seen in the Hi-C contact matrix (Figure 1B, Supplementary Figures S8, S9). Both features are predicted by the bottle-brush model (10), in which bases of nested chromatin loops are helically wound around a linear axis, bringing regions of the genome at a full turn's distance into proximity (Supplementary Figure S8C). Since a similar pattern is also seen in barley, the helical model may describe the higher-order structure in metaphase chromosomes in both plants and animals. According to the bottle brush model, the increase in the Hi-C contacts in metaphase relative to interphase chromosomes at genomic distances of 500 kb, 3 Mb and 30 Mb (Figures 1C, D), match the sizes of minor loops, major loops and helical turns, respectively. A sliding windows analysis (Supplementary Figures S2, S3, Supplementary Movie S1) along all barley chromosomes revealed that the position of the local maximum in the P_C_ plot varied continuously in a range from 20 Mb to 38 Mb, with larger turn sizes in proximal chromosome regions (Figures 1E, F). This calculation allowed us to predict how many turns make up an entire chromosome or smaller regions of it. We found that barley chromosomes ranging from 522 to 675 Mb comprise 18–23 turns, in a positive correlation to the chromosome length (Supplementary Figure S4). Comparing the size of mitotic chromosomes with the expected number of turns, we predict the pitch of each turn to be ∼450 nm.

Plotting helical turn length along the chromosomes (Figure 1E, Supplementary Figure S4) showed several abrupt peaks, which most probably reflect assembly errors in the reference genome sequence. Beyond these putative artifacts, the predicted helical turn length drops dramatically at centromeres and nucleolus organizing regions. These functional chromosomal domains are distinguished by protein complexes bound to the chromatin fibre, which hinder chromatin contacts and complicate the interpretation of a helical arrangement in these regions.

Some animal studies have suggested that helical coiling can only manifest after the overcondensation of metaphase chromosomes, which was seen after a prolonged metaphase arrest brought on by the use of anti-microtubule agents (9,55,56). In our Hi-C study, we applied a 2-h treatment by 2.5 μmol/l amiprophos methyl (APM) to arrest the cells in metaphase. Previous Hi-C studies, conducted on human and chicken mitotic chromosomes (10,17), assessed the effect of nocodazole on chromosome morphology and chromatin contact frequency and distribution. Only a mild effect of the metaphase arrest was observed if the anti-microtubule drug was applied for a short time (<3 h). This indicates that the 2-h APM treatment did not pose a serious obstacle to obtaining a close-to-native picture of chromosome topology. For oligo-FISH, the chromosomes were pretreated 24 h with ice water to accumulate metaphases, a procedure that should not change the higher-order chromatin organization.

### Oligo-FISH confirms the helical organization of mitotic chromatids

We reasoned that *in situ* painting of neighbouring chromosome regions in different colours would show multiple adjacent turns of the helix (Supplementary Figure S6A). To do so, we used fluorescence *in situ* hybridization with pooled oligonucleotide probes (oligo-FISH). Six pools of single-copy 45-nucleotide oligos, covering in total a ∼157 Mb region in the distal part of the long arm of barley chromosome 5H, were synthesized and named after birds for easy reference (Figure 2A, Supplementary Figures S6B–D, S10). FISH with the oligo pools resulted in fluorescent bands of alternating colours across metaphase chromatids as observed using three-dimensional structured illumination microscopy (3D-SIM) (Figure 2B–D, Supplementary Movies S2–6). The visualized bands corresponded to adjacent helical turns (Supplementary Table S3). Adjacent differentially labeled bands partially overlapped, probably due to the stochastic nature of lower-order chromatin loops (Supplementary Figure S10). The height of the labeled bands correlated with the turn length for their corresponding genomic region as determined from Hi-C data (Figures 2E–G). Signals of Stork and Eagle probes, each spanning incomplete turns (about three-fifths of a ∼30 Mb turn), had a mean height of 380 nm (Supplementary Figure S11A). The longer probes Ostrich, Rhea and Moa, each spanning about one and a half turn, gave signals of 460–480 nm height, despite the use of fluorophores offering different SIM lateral resolutions (∼120 nm for Alexa488 and ∼140 nm Atto594). With the observed turn heights, we extrapolate the entire 5H chromosome (to which Hi-C data predict 21 turns) to be 8.0 to 9.4 μm long, which is consistent with microscopic measurements of chromosome 5H length (Supplementary Figure S11A). These findings are concordant with the presence of a helically coiled ∼400 nm thick chromatin thread, which we identify as the chromonema, constituted of chromatin loops.

To determine the volume of the oligo-FISH signals as a proxy of helical turn size, we surface-rendered the 3D-SIM image stacks (Figures 2C, D). To avoid an artificial flattening of chromosomes after preparation, we embedded the isolated chromosomes in polyacrylamide and imaged them by 3D-SIM after DAPI staining. These spatially preserved chromosomes showed ∼1.2-fold higher volumes than chromosomes that were flattened on slides for subsequent FISH (Supplementary Table S4). The entire 5H chromatid, containing 600 Mb of DNA, extended over ∼24.75 μm^3^, corresponding to a density of 1 Mb chromatin per 0.041 μm^3^. The volume of the proximal probes was proportional to their DNA contents, thus chromatin is packed at similar densities in these regions (Figure 2H, Supplementary Figure S11B). Compared to the chromatin density of the entire chromosome 5H, our probe density measurements are too high, possibly because it is difficult to designate single volumes to the sparsely labeled single-copy oligos. Flamingo, the most distal signal, was 1.7 times less dense than the other probes; the only hybridization signal with a volume larger than expected for the targeted chromosome region (Figure 2H). This suggests that the chromatin is more loosely packed at distal chromosome regions, where also smaller helical turns were inferred from Hi-C data. Less compact chromatin may be more flexible. The flexibility of the chromatin arrangement at the chromosome ends was also visible after FISH with subtelomeric and telomeric probes. Their positions varied at the chromosomal termini, and the subtelomeres showed ring-like hybridization signals, as described by Schubert et al. (57) (Figure 2I; Movie 6).

In the oligo-FISH-labeled regions of both mitotic barley 5H chromatids, we always observed the turning of the chromonema with the same handedness (Supplementary Figure S12). In contrast, sister chromatids of human HeLa chromosomes have predominantly opposite (mirrored) helical handedness (55). Because sister chromatids start to separate at the beginning of prophase (58), the turning direction must already be determined after replication during the G2 phase, but it seems to be unrelated to the left-handedness of chromatin fibres, as reported for the salamander *Necturus* (59). Based on investigations of *Drosophila* polytene chromosomes, Sorsa (60) suggested that the size and accumulation of chromomeric loops cause the chromonema to bend and form a helix. In the plant genera *Trillium*, *Rhoeo*, *Osmunda* and *Vicia*, the turning direction can change at the centromere and in different regions along the arms, suggesting that no uniform control mechanism functions throughout the whole chromosome (3,61,62). The ratio of left-handed to right-handed turning appears to be random. Only in certain *Tradescantia* genotypes an excess of right-handed segments point to a specific genetic control (20,63–66). In short, it appears that the higher-order helical turning direction of chromonemata is flexible rather than strictly determined.

The spatial organization of DAPI-stained chromosomes was observed by 3D-SIM (67) to reveal a network of globular, clustered and looped chromatin fibres of ∼80 nm diameter (*n* = 36; mean 77.2 ± 7.7), which possibly corresponds to lower-order chromatin loops, of which the chromonema is composed (Supplementary Figure S13). Complete image stacks revealed chromatin-free regions not larger than 120 × 220 nm (Supplementary Movies S7, S8). At the centromere and nucleolus organizer region, we saw several parallel thin and straight fibres, pointing to a different chromatin organization at these loci, details which remain to be elucidated.

### Sister chromatid exchanges confirm the helical 400 nm chromonema structure

**Figure 3.**
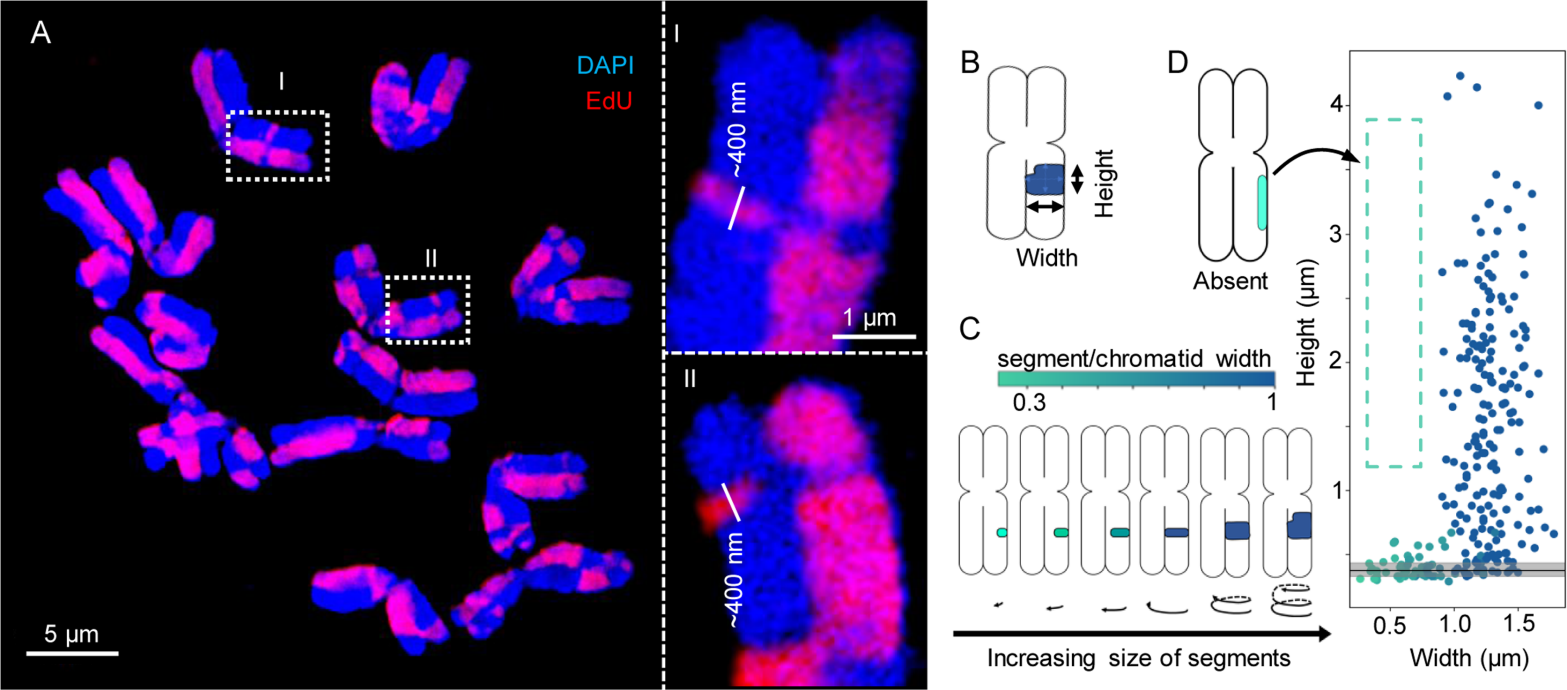
Sister chromatid exchanges (SCEs) confirm the helical organization of barley metaphase chromosomes. (**A**) Metaphase chromosomes labeled with EdU to detect SCEs creating a harlequin pattern. The exchanged chromatin regions appear as thin bands with a minimum height of ∼400 nm (enlarged regions I and II in dashed rectangles). (**B**) Example of the height and width measurements taken from the exchanged segments. (**C**) The colour bar indicates the ratio between the exchanged segments and the chromatid widths. (**D**) The height and width of 257 measured exchanged segments. The lack of values in the dashed region indicates that exchanged segments higher than ∼400 nm and not covering the complete chromatid width (left) never occurred. The black line indicates the median height (∼380 nm) of exchanged segments with incomplete width (less than chromatid width), and the grey area spans the lower and upper quartiles.

**Figure 4.**
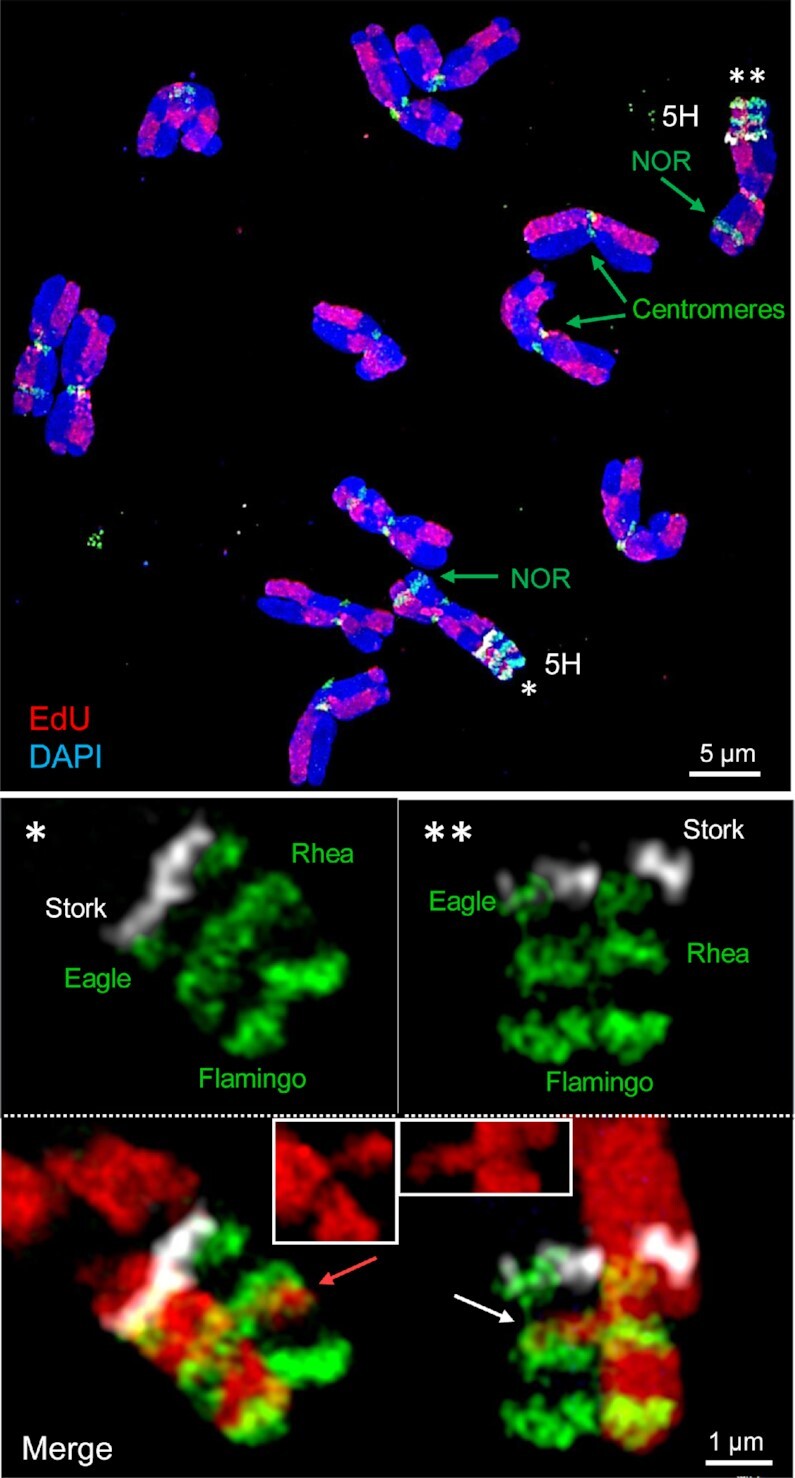
A combination of oligo-FISH and EdU labeled SCEs endorse the helical chromatin organization. Metaphase chromosome spread (top) showing all barley chromosomes after EdU and oligo-FISH labeling. In both homologous oligo-FISH painted 5HL regions two SCEs occurred (bottom). The left exchanged segment does not span the entire chromatid width, but the right one does (insets in the merged images). The SCEs are present at the transition from the Rhea region to the not labeled Moa (left, red arrow) and Ostrich (right, white arrow) regions. The heights of the exchanged segments, oligo-FISH probe and EdU-free regions are similar (∼380–450 nm).

The spontaneous exchange between sister chromatids (SCEs) gave rise to differentially labeled chromatid segments after EdU incorporation during replication, which appeared visually as a harlequin pattern of labeled chromatids (Figures 3A, Supplementary Figure S14, Supplementary Movies S9, S10) (68). We measured the size of exchanged chromatid segments in parallel (height) and perpendicular (width) directions relative to the chromatid axis (Figure 3B). The helical chromonema model puts two constraints on how exchanged segments can be observed by microscopy (Figure 3C). First, exchanged segments spanning more than one helical turn (or more) are as high (or higher) as the chromonema thickness (∼400 nm) and as wide as the entire chromatid width. Second, exchanged segments spanning less than a full helical turn (∼30 Mb) are as high as the chromonema thickness but may not be as wide as the chromatid width. To test these predictions, we observed SCEs in barley metaphase chromosomes using 3D-SIM. The chromosomes differed in the degree of condensation (varying chromatid width and full length), but none of the exchanged segments was smaller than 300 nm in height, approximately the thickness of the chromonema (Figure 3D). Some exchanged segments were as high as the thickness of chromonema but did not span the entire width of the chromatid. Exchanged segments with heights higher than the thickness of the chromonema but not extended over the entire chromatid width did not occur. As soon as the height of the exchanged chromatin exceeded 300–550 nm, it was as wide as the whole chromatid. Next, we combined SCE detection with oligo-FISH, and we found exchanged segments that were coinciding with the FISH signal of the Rhea probe (Figure 4). This confirms that both oligo-FISH and SCEs visualize the chromonema and endorse the predictions of our model.

### Polymer models of helically wound chromatin loops suggest sparse distribution of condensin II complexes

In polymer simulation, we parameterized the bottle-brush model with loops and helical turn sizes as inferred from our Hi-C data (Figures 1G, H; Supplementary Movie S11). In the model suggested for chicken macrochromosomes (10), nested chromatin loops follow a helical path. Loop-extruding condensin II complexes are constrained to this path, mimicking a continuous protein scaffold (Supplementary Figure S8C). Individual condensin II complexes are very close to each other (10 nm), arranging the entire chromatin fibre into densely packed major loops. Each turn of ∼12 Mb is composed of ∼30 major loops filling a pitch of ∼200 nm. In our proposed model for barley chromosomes, each turn (∼30 Mb) has a higher pitch, approximately 400 nm, as verified by microscopy images analysis, and comprised only ten major chromatin loops (∼3 Mb long each). This corresponds to ten condensin II complexes anchored at the loop bases sparsely populating the chromatid axis (Figure 1G) instead of tracing a helical path as in the chicken bottle-brush model. Modelling the barley chromosomes, we assessed different distributions of condensin II complexes, varying the distance between them (Supplementary Figure S15A). After polymer simulations in equilibrium, allowing the bases of the loops to slightly deviate from their original position, we observed that they are better accommodated at larger distances between them (∼100 nm), closely reproducing the observed Hi-C contact probability (Supplementary Figure S15B). Other distance ranges slightly deviated from the observed contact probability, but the characteristic disruption remains, as the chromatin loops are still helically arranged around the axis.

To verify the absence of an axis-forming condensin scaffold proposed for animal metaphase chromosomes (10), we analysed the distribution of condensins within barley chromosomes by 3D-SIM after immunolabeling of the condensin subunit SMC2A. While condensins accumulated at the centromeres, a dispersed distribution and not a continuous scaffold was observed along the chromosome arms. (Supplementary Figure S16).

In the barley model, we noticed that larger spacing between condensin II complexes correlates with longer chromatin loops (Supplementary Figure S15B). We suppose that the folding of big chromatin loops could oppose or stall the extrusion motion of a neighbour condensin complex, leading to loop-free chromatin regions. Single-molecule experiments report that condensins stall upon relatively small opposing forces (69). Previous observations of human metaphase chromosomes with super-resolution microscopy and micromanipulation of chromosome mechanics report that condensin complexes do not form a continuous and uniform helical scaffold (70,71), and we suggest that the chromatids can coil without it.

The sparser and irregular loading of condensin II, also suggests that the major loops may play a smaller role in barley compared to minor loops, held by condensin I complexes. This hypothesis is supported by the dispensability of condensin II (but not condensin I), manifested by recurrent losses of the whole complex or its subunits during eukaryote evolution (reviewed by Beseda *et al.* and Hoencamp *et al.* (2,72)). Hoencamp *et al.* (72) also demonstrated that depletion of condensin II during mitosis in human HCT116 cells induced transition in the interphase 3D genome architecture from type II (chromosome territories) to type I (Rabl-like configurations), typical for barley, other plants and insects, indicating substantial differences in chromatin arrangement between plants and vertebrates driven by different condensin II amount.

Recently, Chu et al. proposed a half-helical arrangement where chromatids change handedness every half-turn (16). To understand the implications of this model on contact probabilities observable in Hi-C experiments, we performed polymer simulations with a coarse-grained model representing a 10-nm thick chromatin fibre, assuming a short turn size. In the half-helical model, regions of the genome separated by half a turn length are either close to each other or far apart, whereas in the helical model they are always far apart (Supplementary Figure S17). Hence, the P_C_ profiles of both models differ markedly, with the helical model resembling both our barley Hi-C data and published chicken data more closely. Half-helical models could be envisioned in different ways. Perversions between half turns were considered, like kinks in a phone cord, but they are very challenging to model. We argue that any model that breaks the helical periodicity would lead to a different contact probability pattern, without the marked decay and increase at a doubled distance.

Apart from the condensins, topoisomerase II (topoII) is one of the major factors shaping the structure of metaphase chromosomes. TopoII tangles or untangles chromatin fibres by cutting and rejoining DNA fibres (73). We considered it in our models, by eventually allowing the chromatid fibre to cross, as proposed by Gibcus *et al.* (10).

### The chromonema coiling mechanism may relate to gene density, chromatin loop length and number of helical turns

We found the predicted turn lengths to be inversely correlated with gene density along the chromosomes (Supplementary Figure S4). This suggests a possible involvement of the epigenomic landscape, mainly post-translational histone modifications, which can locally regulate the structure of mitotic chromosomes (74). For example, the distal end of the long arm of chromosome 5H, which has short turn lengths (Figure 2F), is enriched in genes, genetic recombination (75) and single-copy sequences (Supplementary Figure S6C).

We propose that the chromatin loop length affects the width of the chromonema. In a passive self-coiling mechanism, thinner chromonema sections could lead to locally shorter helical turns. Such a mechanism has been described for other polymers (76,77). It leads to an optimal helical packing, which is consistent with the lack of large cavities that we previously reported for barley mitotic chromosomes (Supplementary Figure S13, (2)). We hypothesize that the breakdown of the nuclear envelope could lead to entropic forces strong enough for the chromatid to coil. Entropy was already suggested to play a role in the dynamics of chromatin (78), but it remains an open question if it suffices to coil the chromatid.

The independent coiling of each sister chromatid poses a conceptual conundrum as discussed by Chu *et al.* (16). As the compaction progresses, the chromosomes would coil into shorter and thicker helices, as indicated by Hi-C data of chicken chromosomes (10). This progressing coiling challenges the cohesion between sister chromatids, held together by cohesin complexes. But we suggest that a mechanism independent of a uniform protein scaffold can accommodate other compelling forces. Cohesins, belonging to SMC protein complexes, work dynamically, possibly binding and unbinding while chromatin loops are stretched and released without affecting the coiling of the chromonema. Likewise, a discontinuous protein scaffold may be consistent with the deformed axis observed by Chu *et al.* (16).

### The chromonema-based model of a mitotic metaphase chromosome

**Figure 5.**
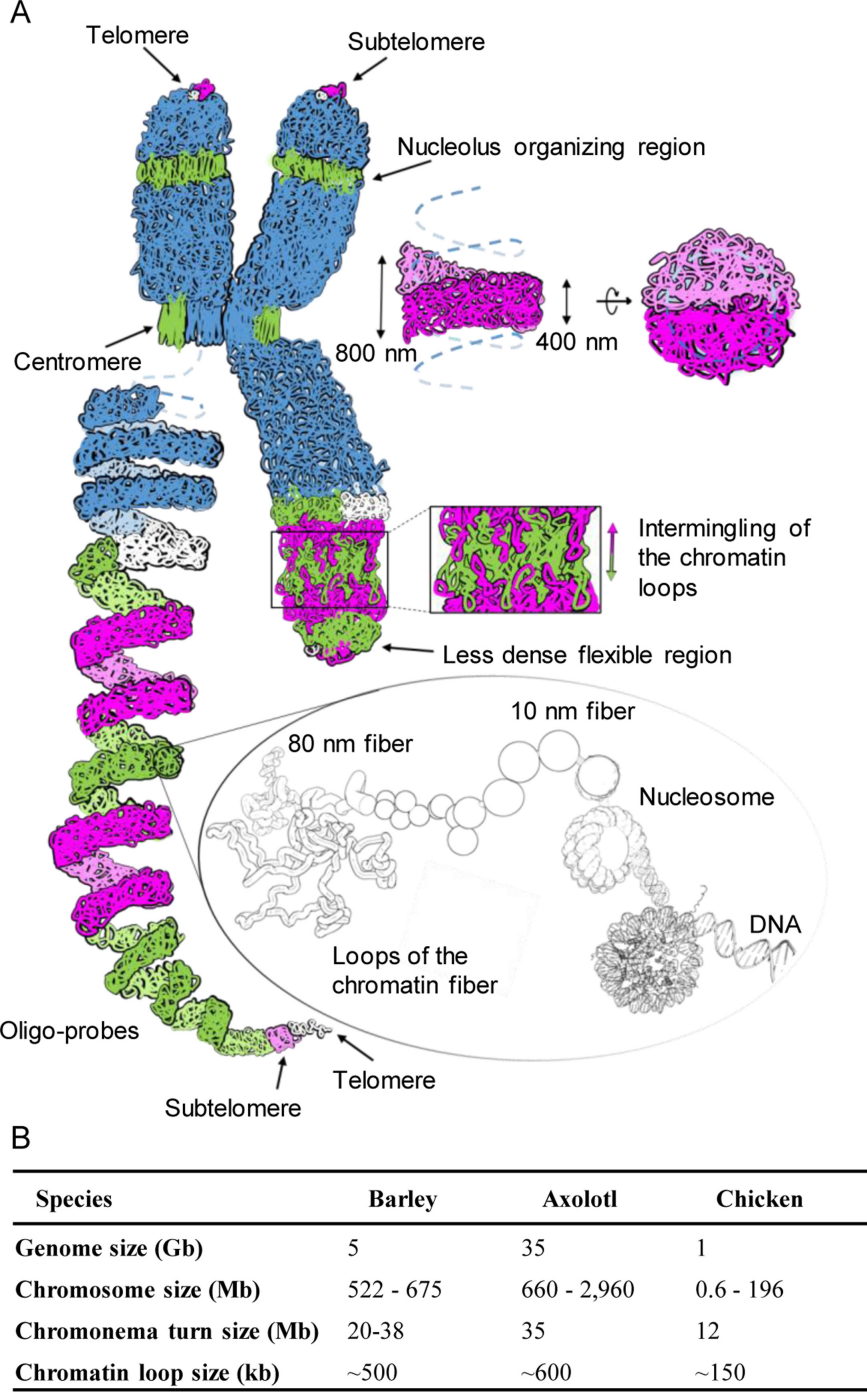
Helical coiling of the chromonema. (**A**) Model of the chromatin organization in barley metaphase chromosomes. Bottom right: loops of the 80 nm lower-order chromatin fibre, formed of consecutive nucleosomes. Consecutive loops of simulated chromatin coloured as in our microscopic observation form the chromonema, which coils fill completely the chromatid without large cavities. Adjacent chromonema turns intermingle at their edges due to the flexibility of the smaller 80 nm fibres present within the chromonema. The helical order is interrupted at centromeres and secondary constrictions displaying mainly straight 80 nm chromatin fibers. The chromosomal termini contain less condensed, more flexible chromatin. Due to this flexibility, the telomeres may be embedded into the subtelomeric chromatin and not appear at the very end of the chromatid. The left long chromatid is shown as a stretched helix representing its higher-order chromatin folding based on oligo-FISH labeling. The different colours represent incomplete (white + green) and complete (magenta + green) chromonema turns, respectively. (**B**) Parameters of helically organized somatic metaphase chromosomes of barley compared to other species. Chromatid helix turn sizes and nested loop sizes are based on Hi-C data.

The microscopic observations reported here and our analysis of Hi-C data combined with polymer simulations support a helical higher-order structure of mitotic chromatids (Figure 5A). We consider the bottle-brush of Gibcus *et al.* (10) and our chromonema as compatible formulations of the same model. Bottle-brush emphasizes chromatin loops emanating from a helically wound basis, while chromonema focuses on the ensuing higher-order entity of a chromatin helix. In our opinion, reviving the classical term chromonema affords greater conceptual clarity to the latter entity, even more so as it is not only a concept but a structure observable under the microscope.

The chromonema coiling enables a further degree of chromatin condensation, possibly required to handle larger chromosomes developed during evolution. To compare the higher-order structure between small and large chromosomes, Kuznetsova *et al.* investigated mitotic chromosomes in a group of plants differing in genome and chromosome sizes using transmission electron microscopy (79). The authors observed large chromatin-free cavities in axial regions of large chromosomes, including those of barley, and proposed a new plant-specific model distinguished by large axial cavities within the chromatids. In our previous (2) and the current study (Supplementary Figure S13, Supplementary Movies S7, S8), we demonstrated by 3D-SIM that the large cavities are likely preparation artefacts and only small chromatin-free regions without a significant impact on chromosome topology are present in barley mitotic chromosomes. However, a detailed analysis of the interphase chromosome arrangement in diverse species revealed fundamental differences between mammals and other organisms, and it was hypothesized that these differences are related to the loading of condensin II during the mitotic division that precedes interphase (72). This logically implies that particular, yet undiscovered organizational patterns of metaphase chromosomes may occur in some systematic groups of species.

Still, the helical organization was reflected in the accurate data gathered for large (>12 Mb) chromosomes of chicken (Supplementary Figure S7C) (10), axolotl (8) and barley (Figure 5B), which also consistently suggest that there is a relationship between the size of the loops and the size of the helical turn. Biopolymers are known to coil due to the entropy of their environment (77). In this process, the geometry of the helix changes according to the width of the polymer (80). Likewise, differences in the chromonema width, dependent on loop sizes, may explain the inter-species differences in helical turn size. A model of a self-coiling mechanism, together with a deeper knowledge of the spatial distribution of condensin complexes, may explain the matter in the future.

## DATA AVAILABILITY

The Hi-C sequencing data are deposited at the European Nucleotide Archive under project ID PRJEB45629 (https://www.ebi.ac.uk/ena/browser/view/PRJEB45629?show=reads).

## Supplementary Material

gkad028_Supplemental_Files
