# Supplementary material for: Helical coiling of metaphase chromatids
Source: Nucleic Acids Res. 2023 Mar 2;51(6):2641–54. doi: 10.1093/nar/gkad028 (PMC10085683; doi:10.1093/nar/gkad028)
Supplement: gkad028_Supplemental_Files [file gkad028_supplemental_files.zip › SupplementaryMaterial_corrected.pdf]

Supplementary material

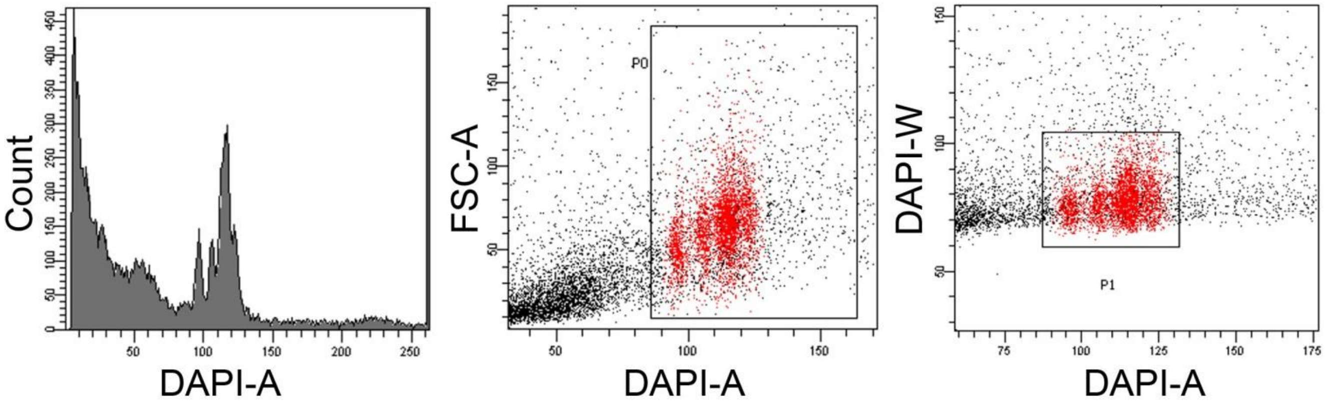

Figure S1. Flow sorting of barley metaphase chromosomes. Flow karyotype of barley cv. Morex. Initial gating was set-on relative DAPI fluorescence vs. forward scatter parameters (P0), dependent sorting gate was drawn on DAPI-area vs. DAPI-width scatter plot (P1).

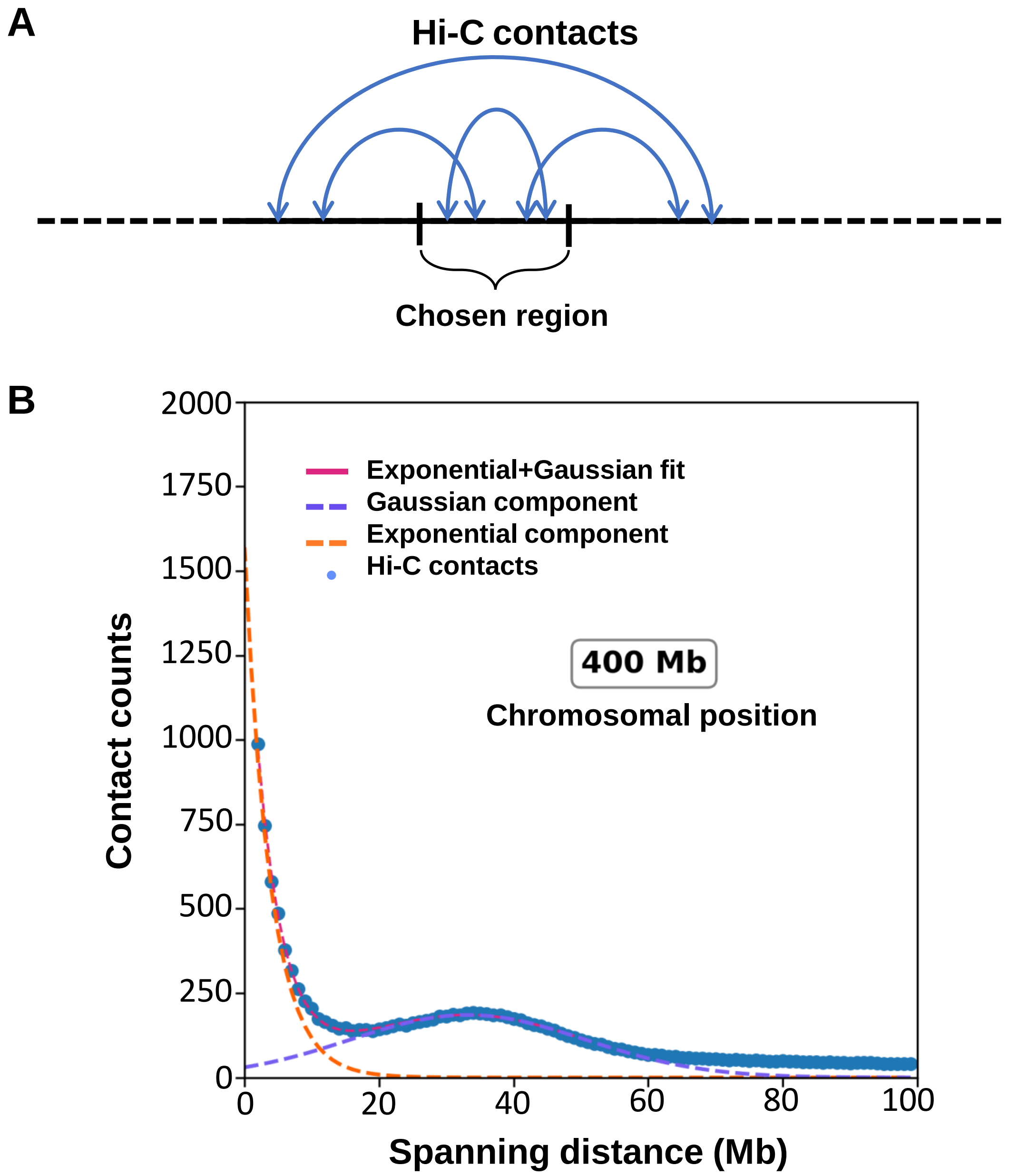

Figure S2. Quantification of Hi-C local contacts in metaphase chromosomes. The chromosomes were divided into 5 Mb-long non-overlapping regions. **(A)** All Hi-C pairs within or spanning each region were analyzed as a function of the distance between the contacting sites. For each region, such a function is plotted as the number of contacts distant by 1 Mb, 2 Mb, ... until 100 Mb **(B)**. Normalized, it can be interpreted as a local contact probability. This function is well described by the sum of an exponential and a Gaussian distribution. The Gaussian distribution coincides with the bump characteristic to a helical arrangement (Fig. 1A). Its center indicates the turn length of the helical arrangement around the analysed region, and it was used to build the graphics in Figs. 1E and S7.

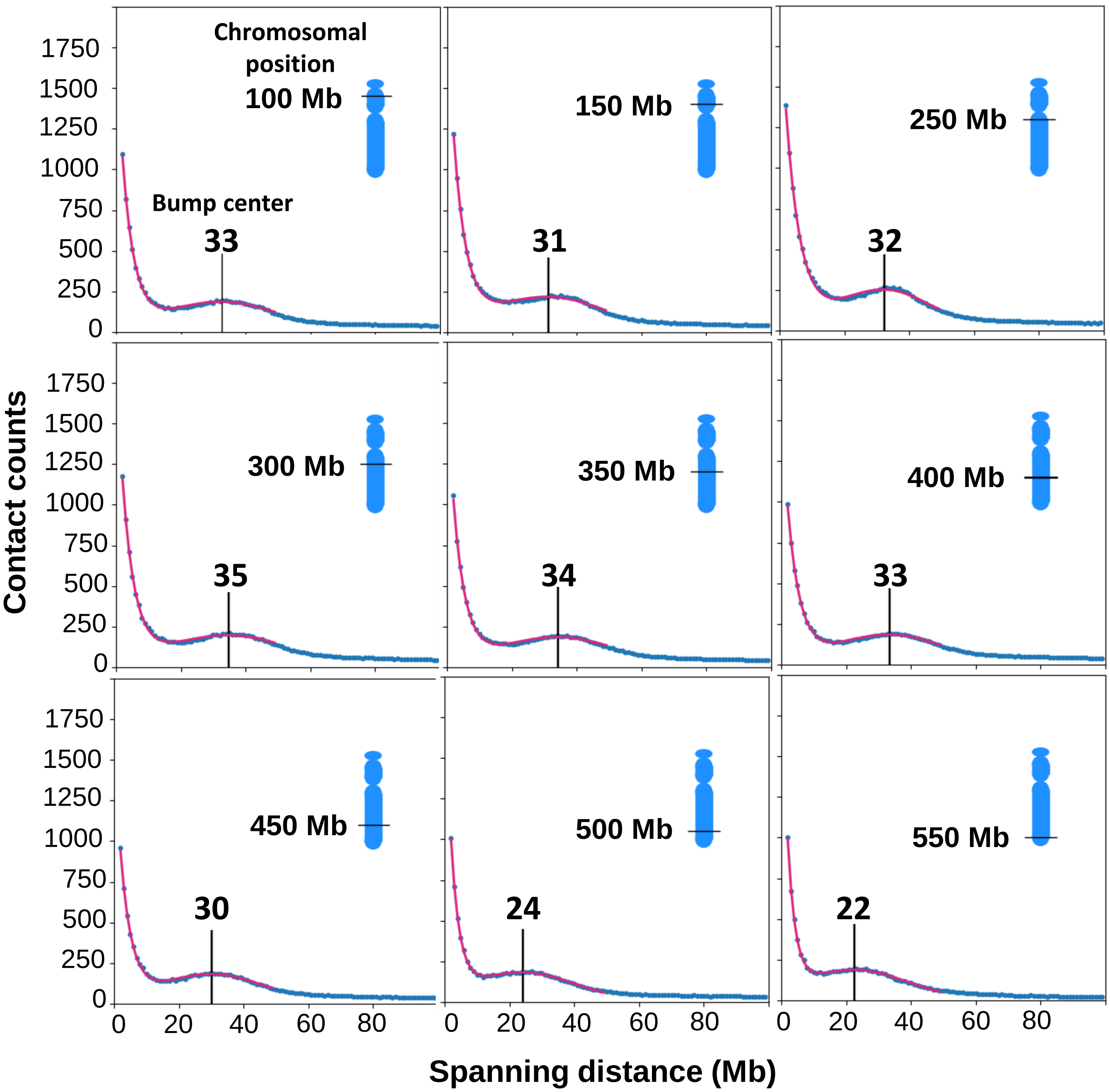

Figure S3. Local contact amounts at nine different positions (100-550 MB) of chromosome 5H retrieved from experimental Hi-C data. At all positions a prominent bump is present, with the center at slightly different positions. The bump represents a contact increase followed by a decrease at half of its position. This pattern indicates the chromatin arrangement into turns, at which the turn length corresponds to the center of the bump. The bump position at any region along chromosome 5H has been calculated as described in Fig. S2. The placement of the bump for all 5 Mb regions (119 regions in total), separated by 5 Mb along the 5H sequence is visualized in Movie S1.

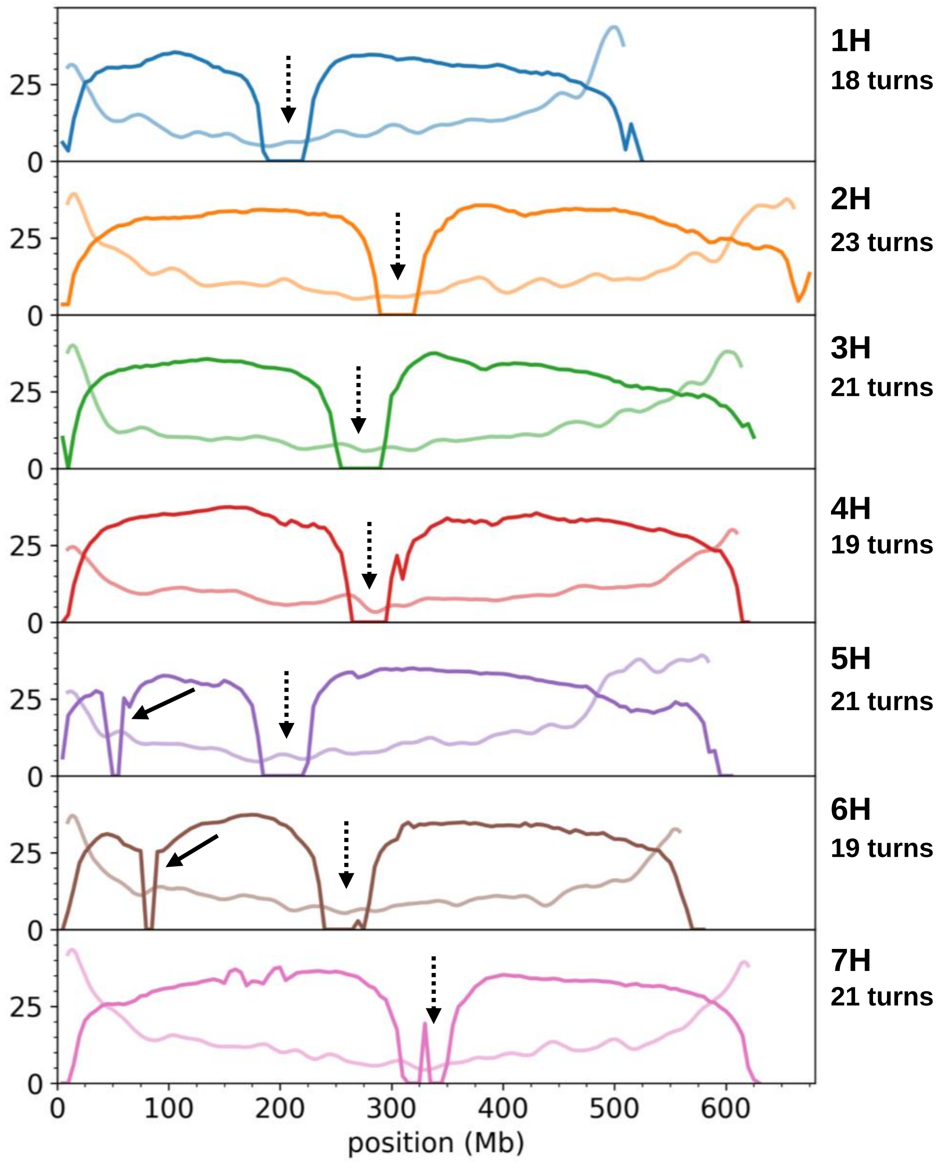

Figure S4. Turn length (Mb) (Table S2) based on the local contacts observed in the Hi-C experiments and gene density (number of genes per Mb) along all chromosomes. Values for each chromosome are differently colored, as in Figure 1E. Turn length is indicated by the solid line and gene density by the transparent line. Values on the vertical axis are valid for both turn length (Mb) and gene density (number of genes per Mb). We observe an inverse correlation between turn length and gene density. Positions of primary (dashed arrows) and secondary (solid arrows) chromosome constrictions can be recognized by zero turn length. All interstitial regions show similar turn lengths, decreasing towards the telomeres. Abrupt peaks in the turn length, such as in chromosome 7H at 160, 195 and 330 Mb, relate to gaps or assembly errors in the genome sequence. The total number of turns per chromosome is indicated on the right.

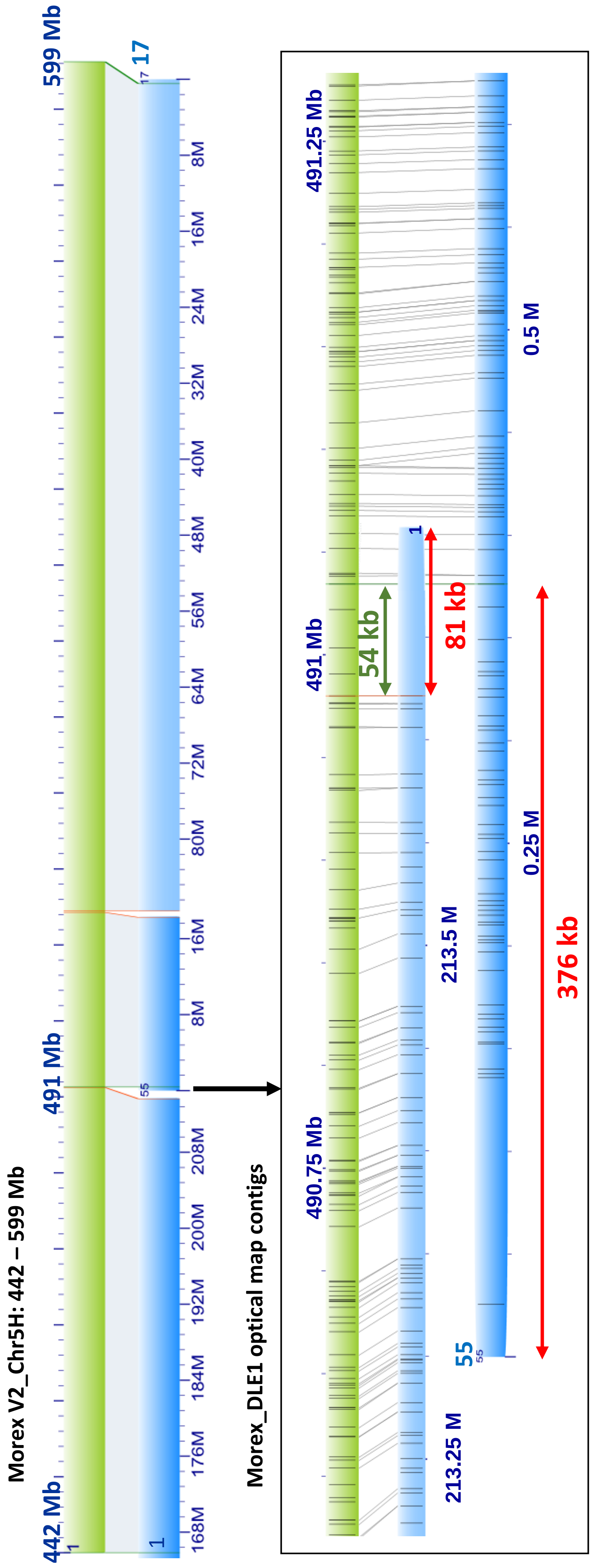

Figure S5. Validation of the chromosome 5HL interval 442-599 Mb in the Morex V2 genome assembly by optical mapping. The selected sequence segment (green bars) shows a good alignment to the optical map contigs 1, 55 and 17 (blue bars), indicating overall correctness and completeness of the sequence (top), except a region around 491 Mb where non-aligned overhangs of contigs 1 and 55 indicate a missing sequence with a minimum of 403 kb (bottom).

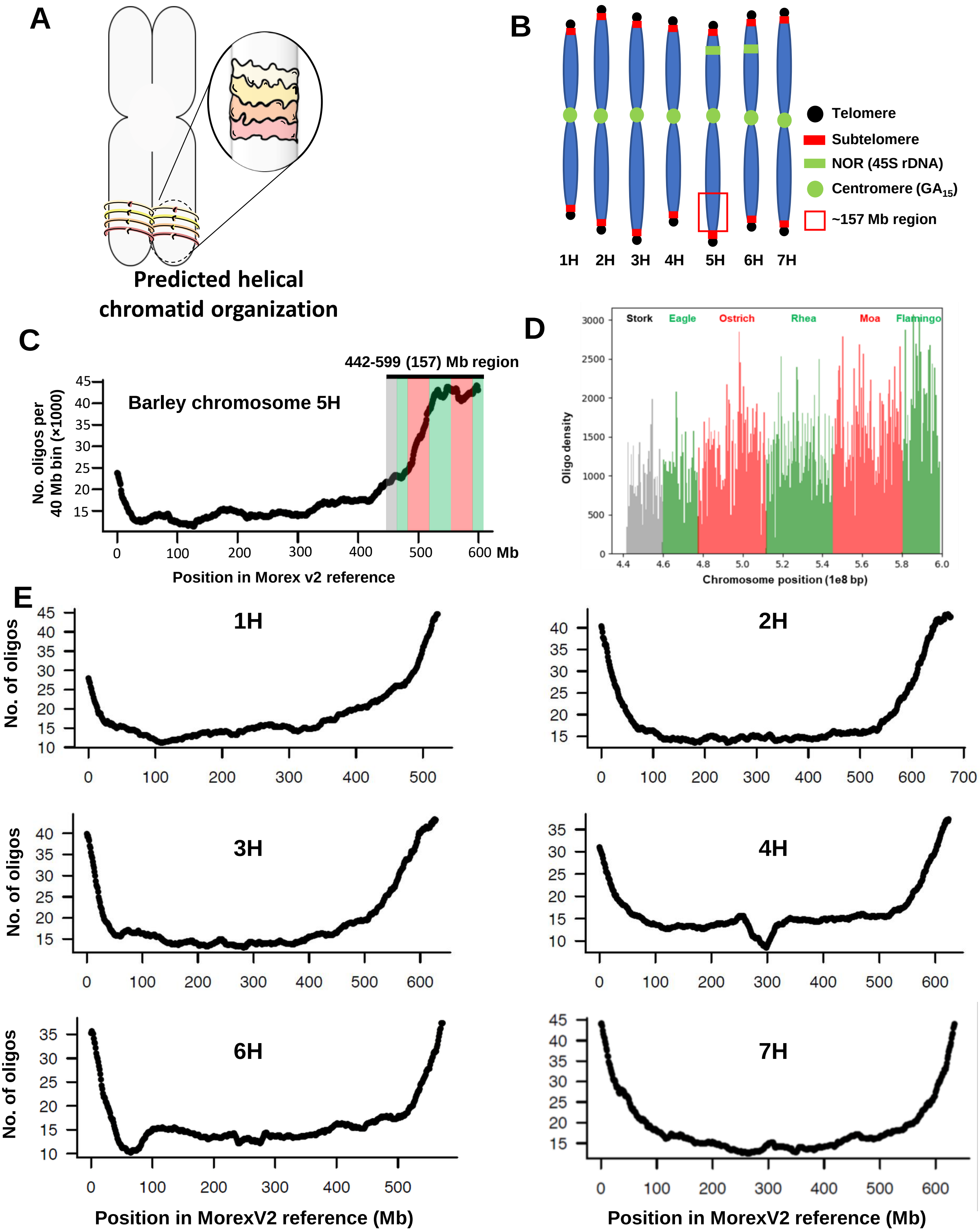

Figure S6. Oligo-probe distribution. **(A)** Schema of a metaphase chromosome arranged into turns as inferred from the Hi-C data. **(B)** Karyogram of barley indicating the distribution of the applied centromere-, NOR-, subtelomere- and telomere-specific FISH probes. **(C)** Density of single copy oligos along chromosome 5H. The distal 157 Mb-long region of the long arm of chromosome 5H is enriched in single-copy sequences suitable for the detection by oligo-FISH. **(D)** Distribution of the oligo-probes at the long arm of chromosome 5H. **(E)** Density of single copy oligos along all barley chromosomes (except 5H shown in **(C)**). The x-axis shows the oligo position at the chromosome in Mb. The y-axis represents the number of oligos within 40 Mb bins.

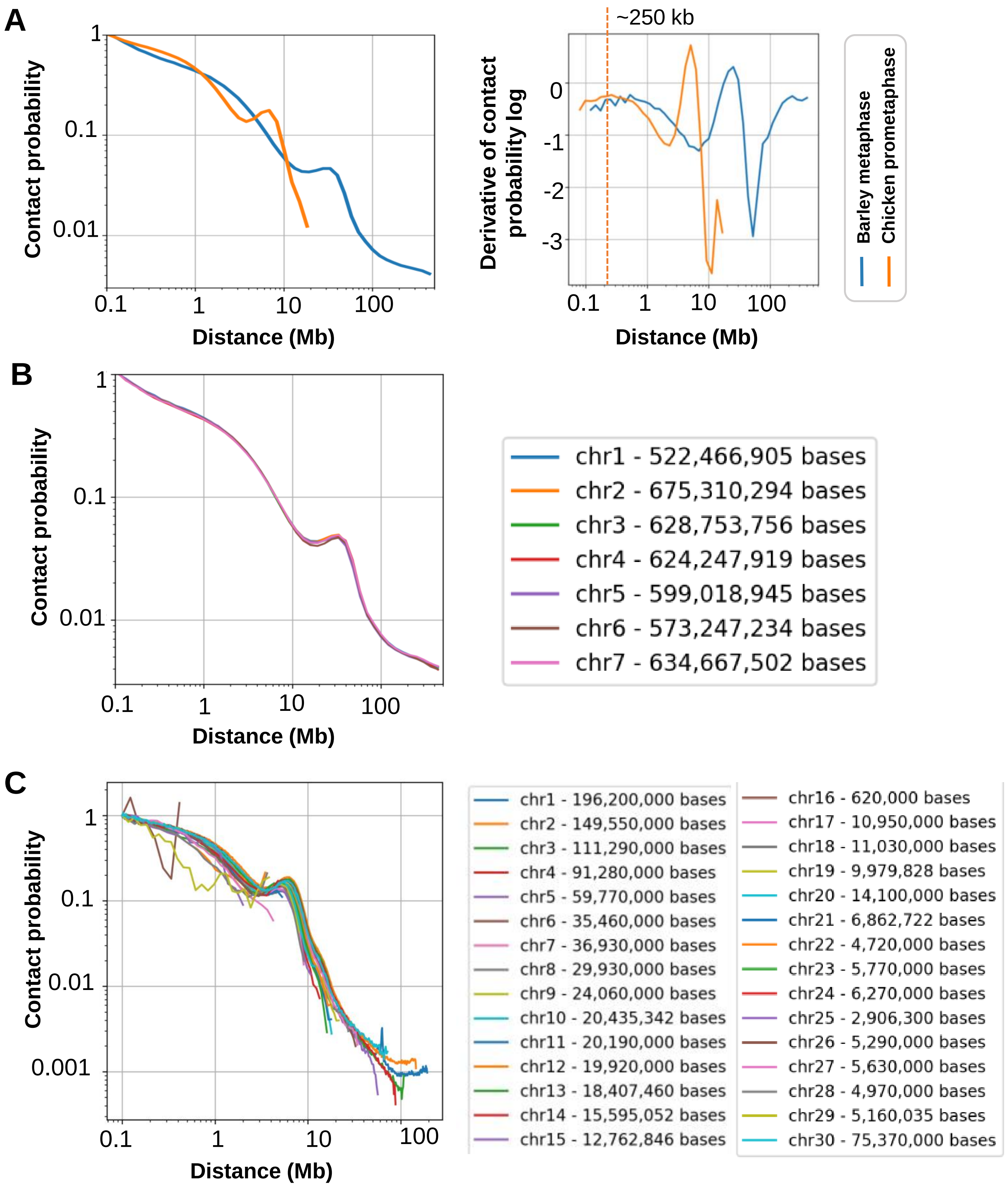

Figure S7. **Comparison between chicken and barley Hi-C based contact probabilities of mitotic chromosomes. (A)** Contact probabilities of barley chromosome 5H at metaphase and chicken chromosome 1 at prometaphase (Gibcus et al., 2018) normalized to be equal to 1 at 100 kb**.** The right diagram shows the derivative of the same contact probabilities. **(B)** Contact probabilities of all barley chromosomes at metaphase and **(C)** of all chicken chromosomes

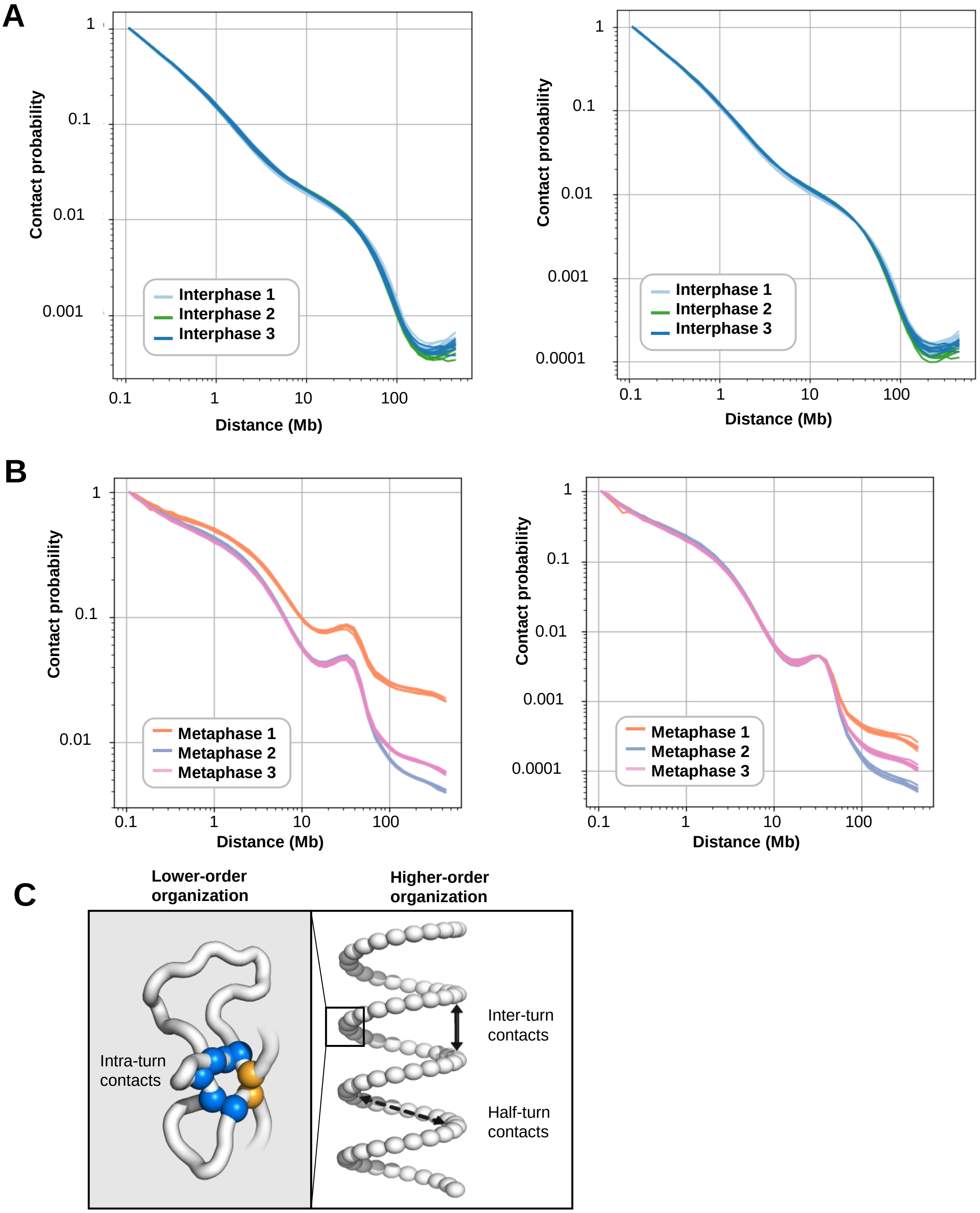

Figure S8. Hi-C contacts in metaphase indicate a helical organization. Contact probabilities calculated from the three Hi-C experiment replicates of interphase **(A)** metaphase **(B)** chromosomes. On the right the probabilities are exponentially normalized to have the same values at 100 kb and 30 Mb. **(C)** The three contact types observed with different contact probability patterns are represented in a helical arrangement. Sites distant by one turn are closer to each other (solid arrow, inter-turn contacts) than sites distant by half-turn (dashed arrow, half-turn contacts), and contacts between adjacent loops (inset) constitute a lower-order organization with higher contact probability. At this level, the chromatin fiber (white) is folded into major and minor loops. Condensin II proteins hold the bases of major loops (yellow). These are further divided into minor loops, whose bases (blue) are held by condensin I complexes.

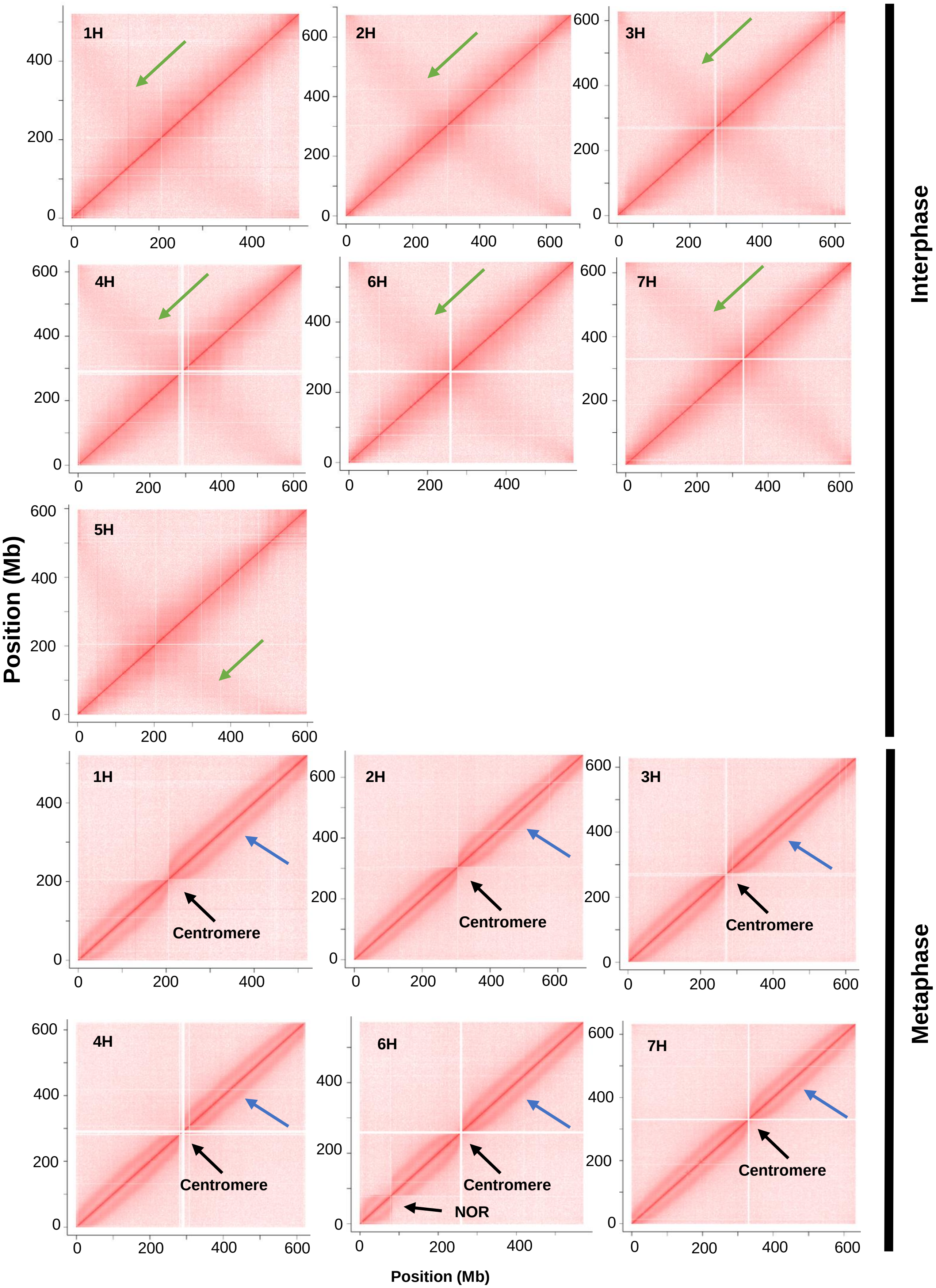

Figure S9. Hi-C contact matrices of all barley chromosomes at inter- and metaphase (except 5H in metaphase shown in Figure 1B) from the first replicate of the Hi-C experiments. The anti-parallel diagonal in interphase (green arrows) indicates the Rabl configuration, while metaphase chromosomes show a parallel diagonal (blue arrows), indicating a periodical contact pattern missing at the NOR of 6H and the centromeres (black arrows).

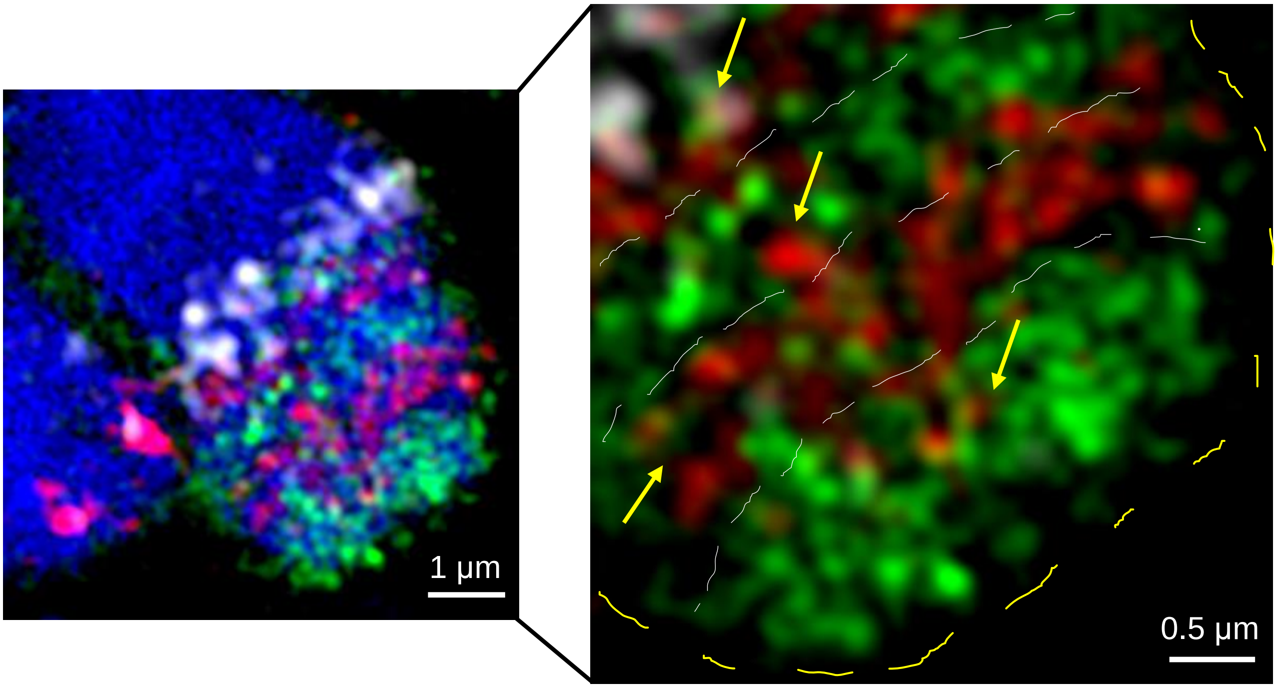

Figure S10. Adjacent helix-turns are partially intermingled. The differentially oligo-FISH labeled turns at chromosome arm 5HL intermingle with up to ~50% of their thickness. Smaller looped chromatin fibers forming the turned chromonema (indicated by dashed lines) and marked by the different colors invade into each other (arrows in the right enlarged image). Chromatin was counterstained with DAPI (blue).

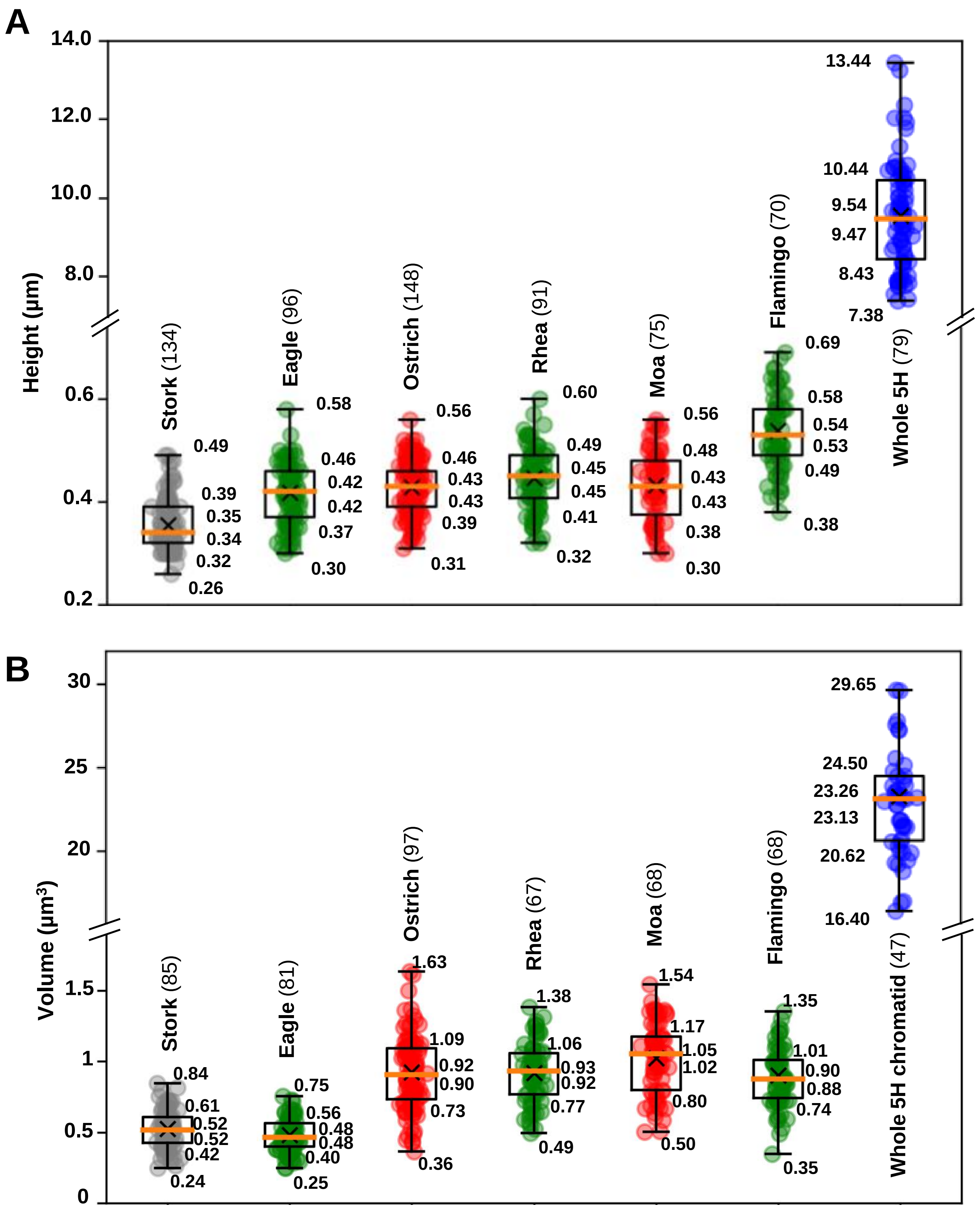

Figure S11. Box-plots showing the measured signal heights (A) and volumes (B) of the oligo-labeled regions on chromosome 5HL. The variable heights of the probes are consistent with the different turn lengths along the mitotic chromosome (Fig. 2E) and the different metaphase chromosome condensation degrees. Volumes were measured after surface rendering using Imaris 9.6, and the values correspond to a single chromatid. The numbers of measured chromosomes per oligo probe are in parentheses.

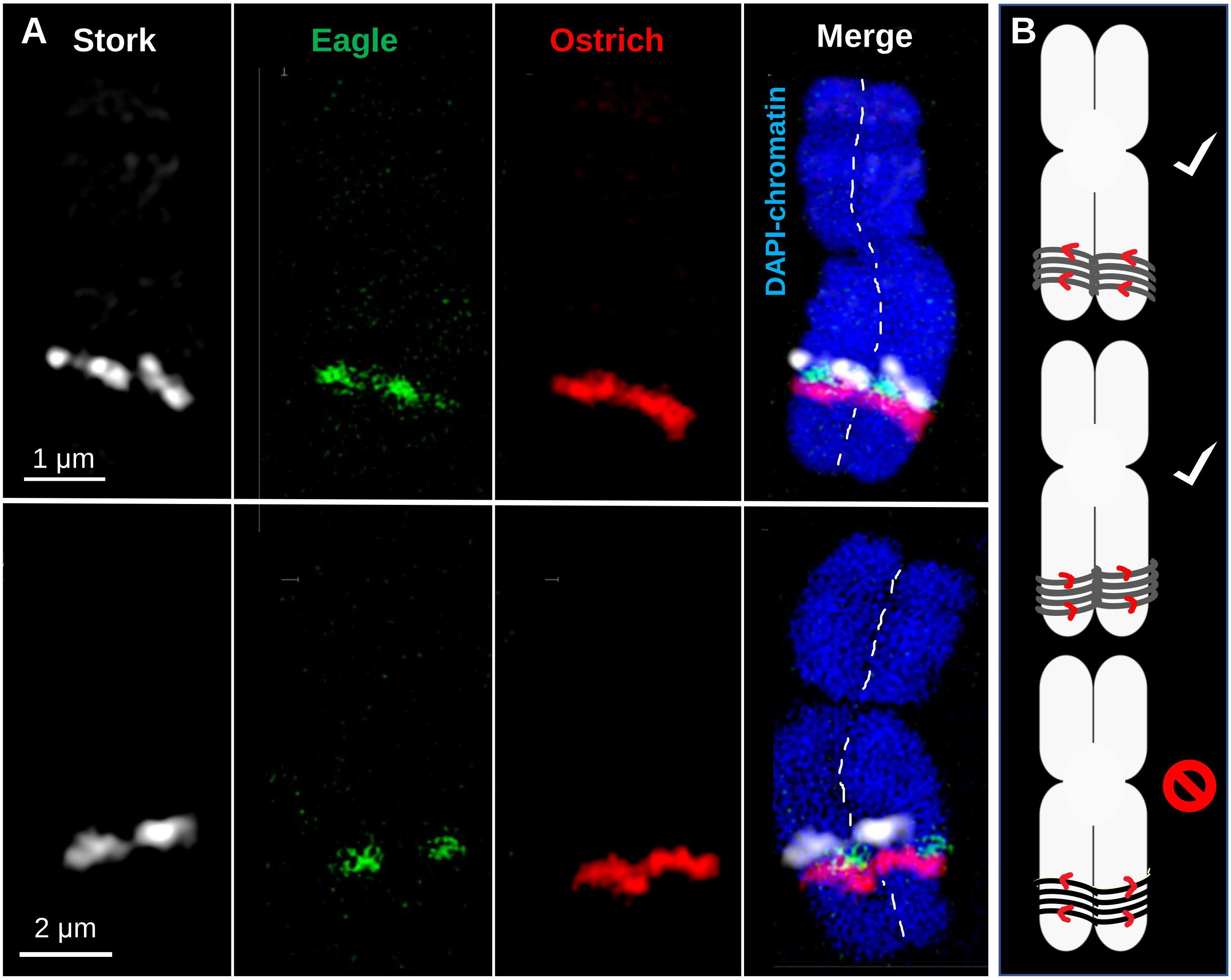

Figure S12. In both chromatids the ~430 nm fibers turn in the same direction. (A) The oligo-FISH probes (here two examples of Stork, Eagle and Ostrich) reveal that the sister chromatids coil in the same direction, and not in a mirrored manner. In the merged image the sister chromatids are divided by a dashed line. **(B)** The schemata show the observed (top and middle) and not occurring (bottom) arrangements.

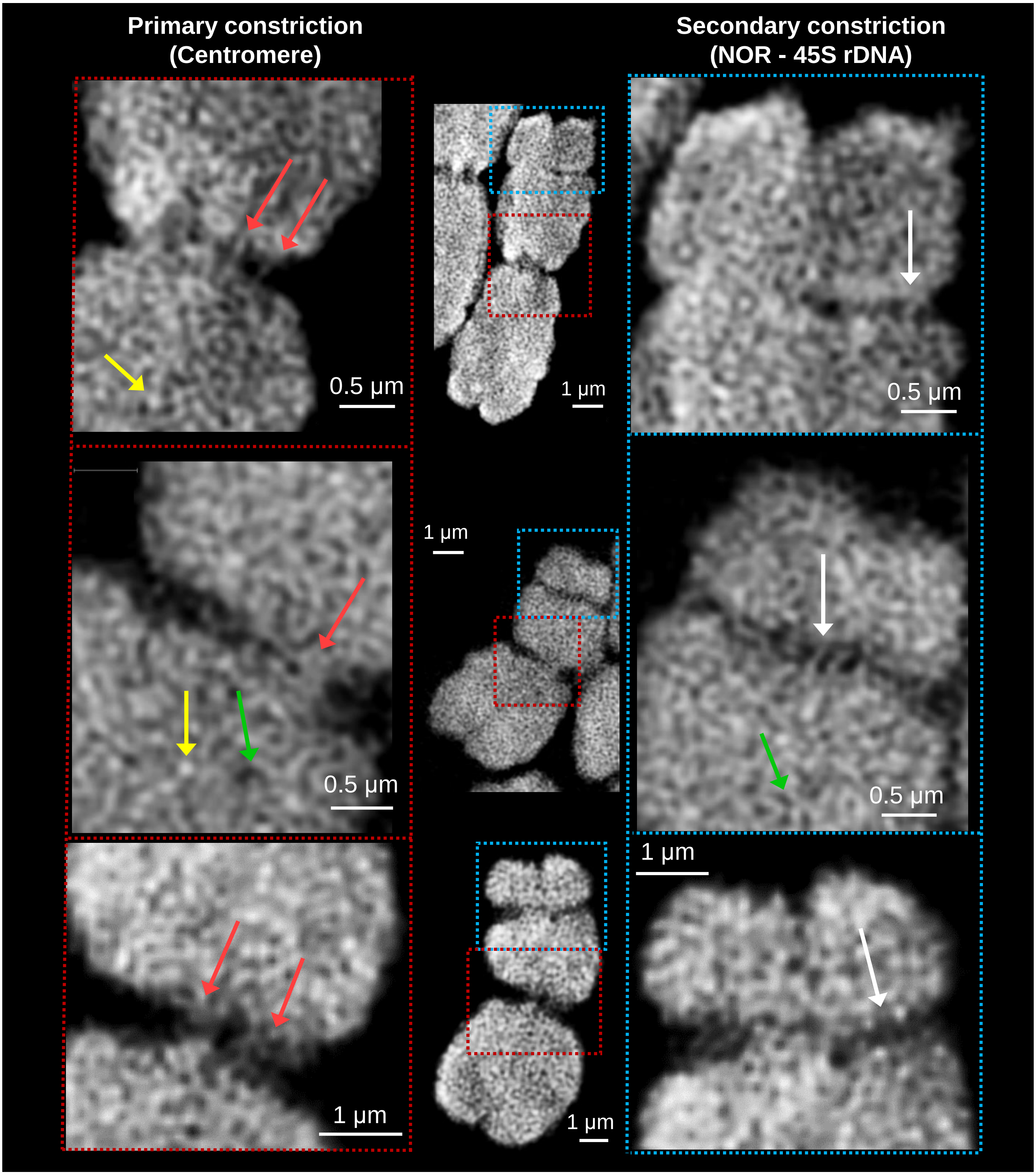

Figure S13. Primary (centromere) and secondary (NOR) chromosome constrictions are not organized as a spiral. Instead, these regions (marked by red and blue dashed rectangles) contain parallel chromatin fibers (red and white arrows) (see also Fig. S14A). At chromosome arms, the DAPI-labeled chromatin (white) occurs as a network of looped fibers within the ~430 nm fiber (here not separately visualized by oligo-FISH or SCEs). The fibers contain chromatin accumulations (yellow arrows), and in between them, small chromatin-free regions (green arrows). No large cavities were observed as demonstrated also in Movies S7, S8 representing a whole chromosome volume.

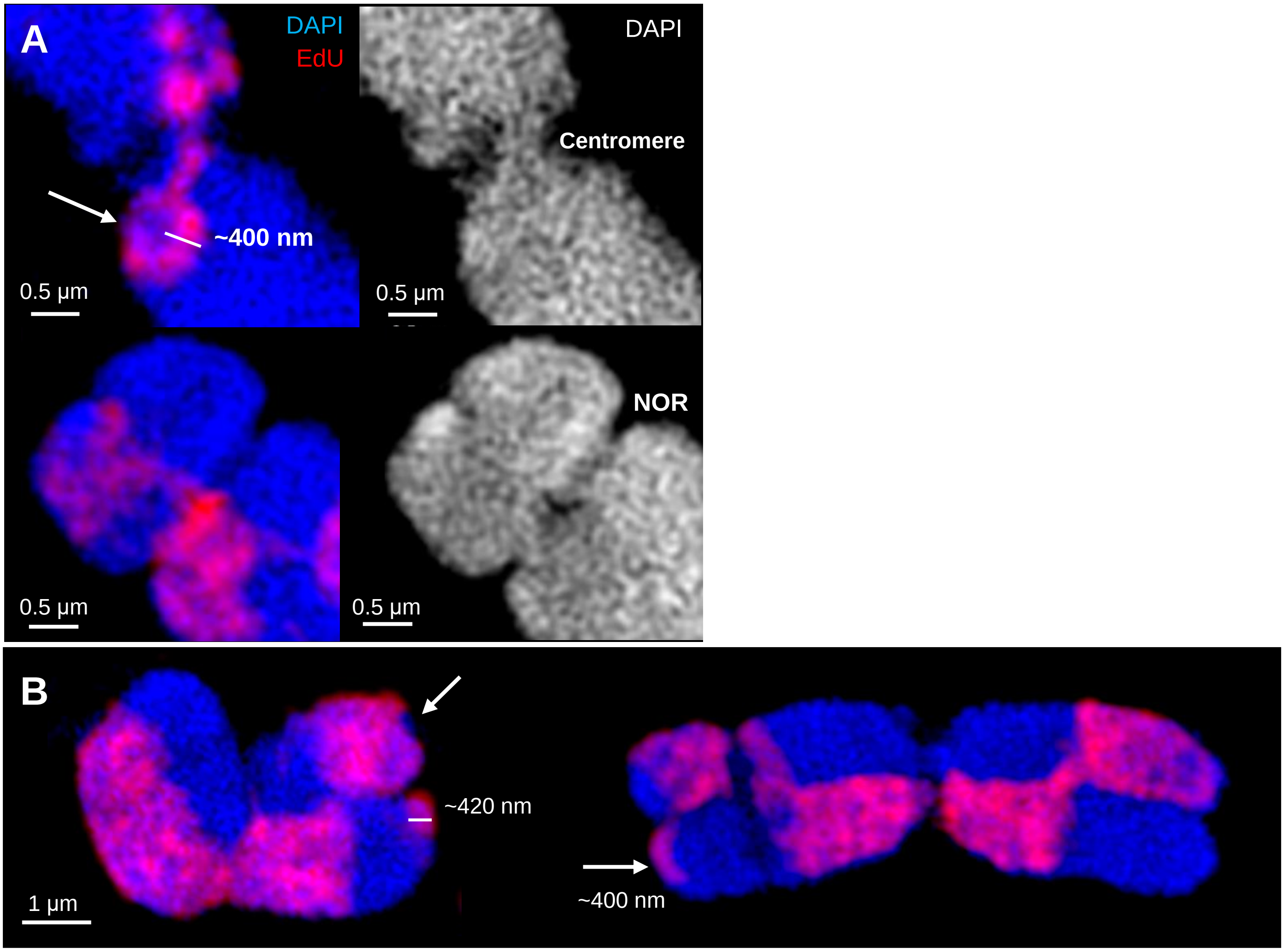

Figure S14. Sister chromatid exchanges (SCEs) at centromere and telomeres. (A) At centromeres (top) the EdU labeling indicates a straight thin connection between the ~400 nm high fibers. Immediately next to the centromere, due to chromatin tilting, a ~400 nm thick turn is visible in the pericentromeric region (arrow). At the NOR (bottom) also thin chromatin fibers occur. **(B)** Exchanged segment units of ~400 nm are also evident at the chromosomal termini (arrows). Chromatin was counterstained by DAPI (blue/white).

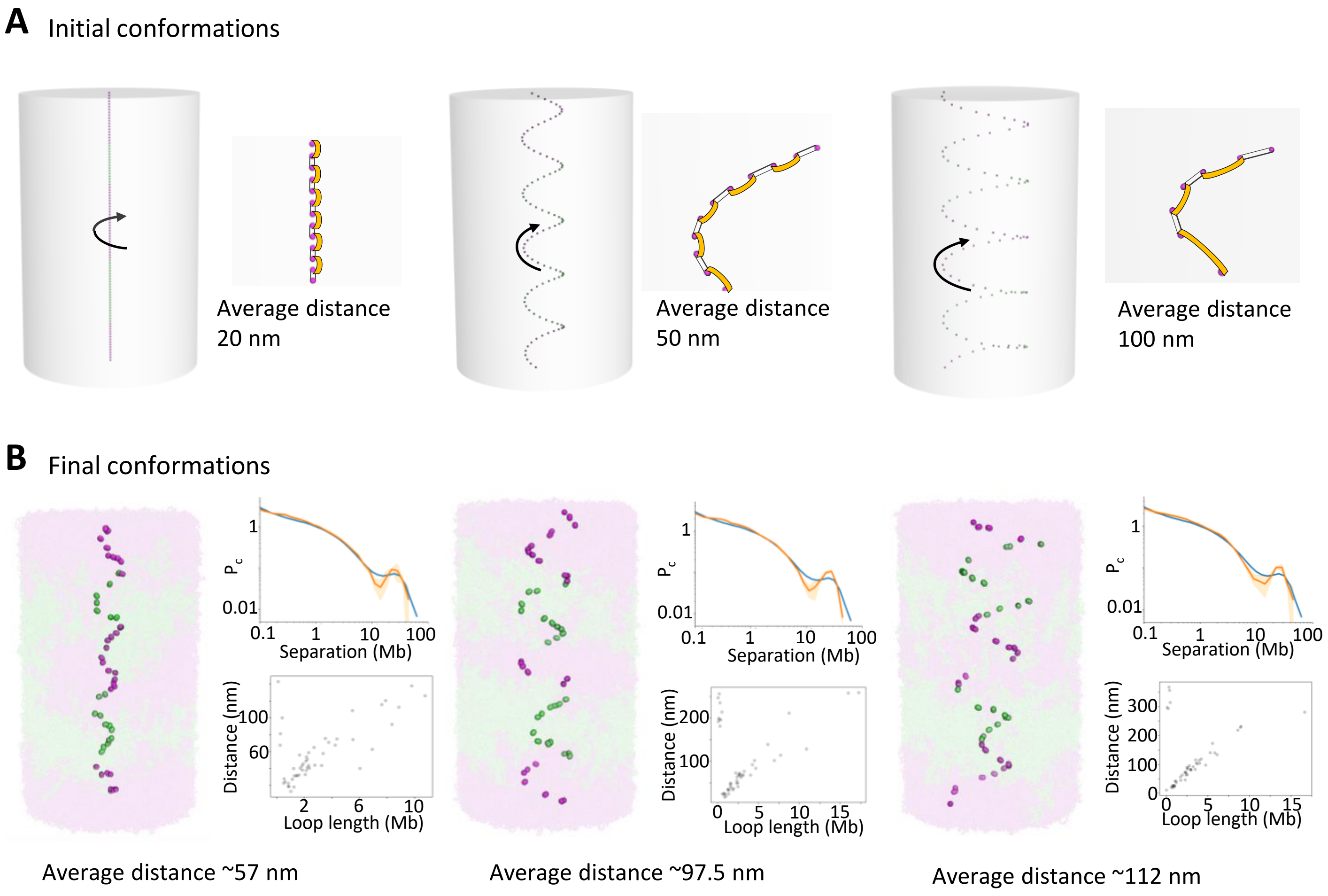

Figure S15. Models for loop spacing. As proposed by Gibcus et al. (2018), these models consider that there is a condensin II complex holding a pair of nucleosomes at the basis of each chromatin loop. The bases of the chromatin loops are tethered side by side to a helical path, with no interspaced nucleosomes. The nucleosomes at the loop bases may deviate from the starting position and stretch the distance between each other during the simulation, while accommodating the loops inside the cylinder. We present three models, differing in the initial distance between the anchored nucleosomes: 20, 50 or 100 nm. Each nucleosome at the loop basis is shown as a small bead. **(A)** Initial conformation for each model. The helical path (beads), the cylindrical constraint (grey surface), and the helical winding of the chromatin loops (black arrow) are shown on the left. A detail of the loop bases is shown on the right. Yellow rings indicate the pairs of nucleosomes held at the loop bases, and black lines connecting the beads indicate that they are neighbor nucleosomes in the chromatin fiber. 20 nm is the minimum average distance between monomers at the loop bases that supports the parameters for the barley mitotic chromosome (namely, 400 nm pitch, 30 Mb turn, 3 Mb major loops and 500 kb minor loops). With 20 nm between them, the anchored nucleosomes form a line instead of a helical path. At larger distances, the beads wind around the axis forming a helix with larger radius. **(B)** Final conformations of the same models shown in **(A)**. On the left, the transparent chromatin loops fill the constraining cylinder and the anchors loosely follow the initial path. Individual helical turns and the corresponding loop bases are colored alternately in pink and green. Calculated (orange) and experimental (blue) contact probabilities (Pc) are shown on the top right of each model. On the bottom right is a plot for the distance between the loop bases and the length of their corresponding chromatin loops. The final average spacing of loop anchors is indicated below each model.

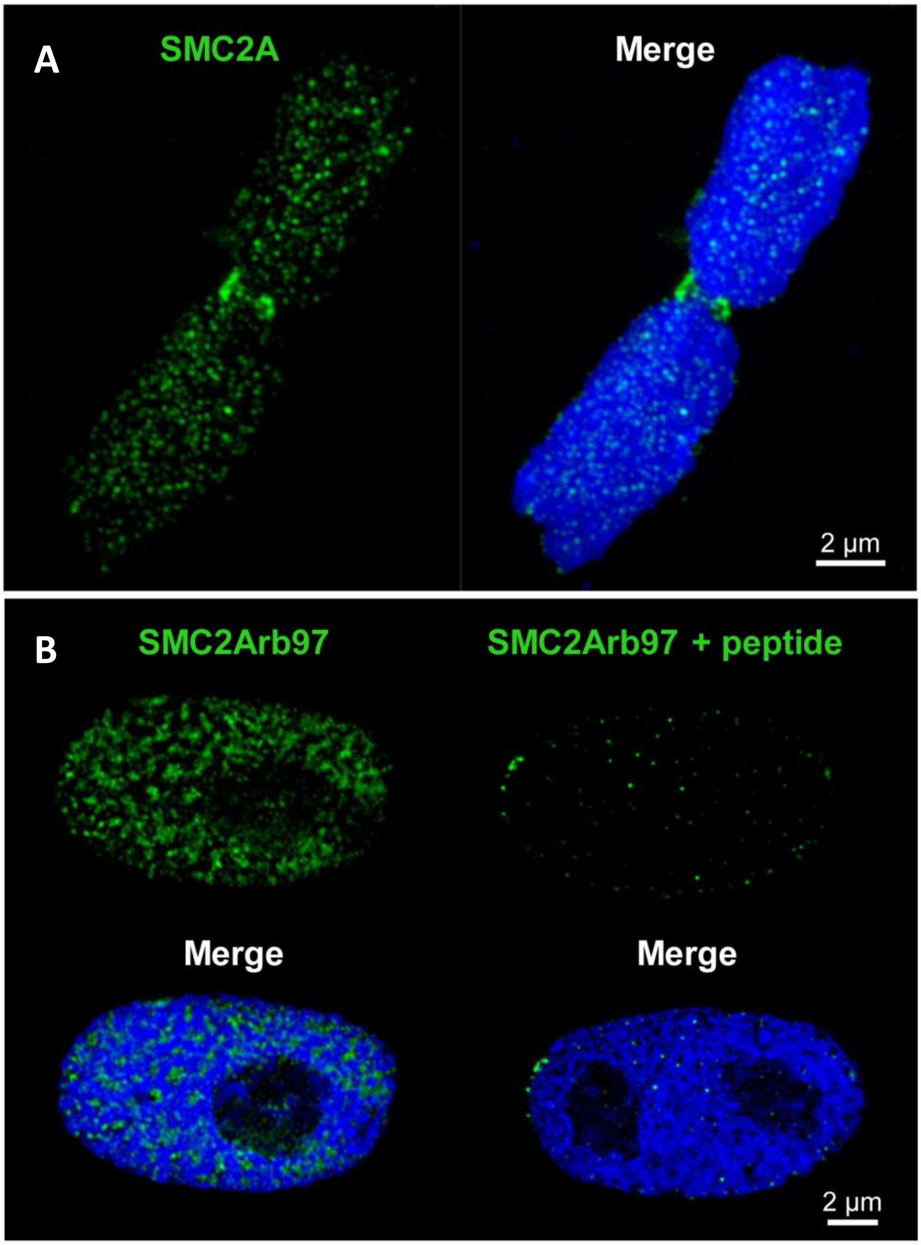

Figure S16. SMC2A immunolabeling in a barley chromosome (A) and proof of the specificity of the antibodies by peptide competition (B). (A) An isolated barley chromosome immunolabeled with SMC2A antibodies shows fluorescence signals accumulated at centromeres and distributed along chromosome arms. **(B)** Immunolabeling of isolated flow–sorted 4C root nuclei without (left) and with peptide competition (right). The addition of the peptides, in 1:10 excess, to the SMC2A antibodies raised in rabbit 97 become specifically sequestered by the corresponding peptide, resulting in a complete competition. No specific fluorescence signals were detected.
Chromatin was stained by DAPI (blue). All images are single slices from 3D-SIM image stacks.

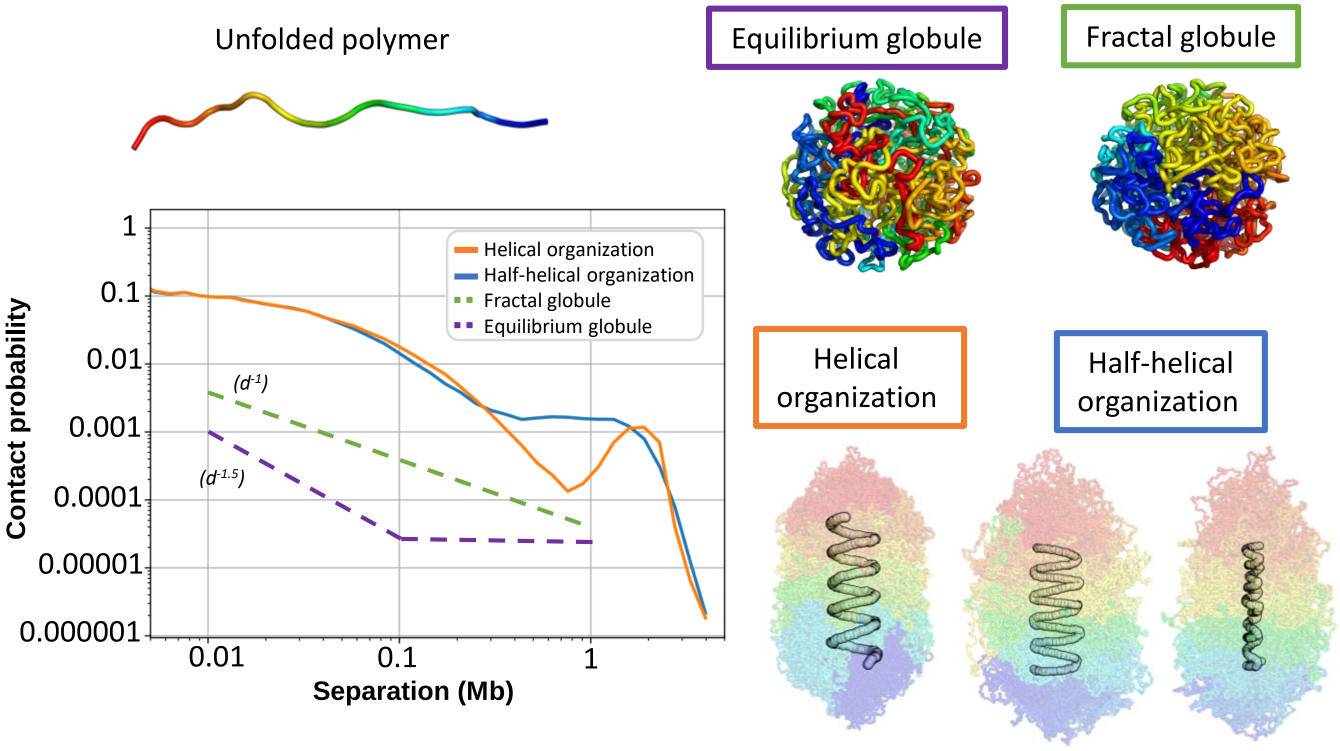

Figure S17. Contact probabilities of polymer models support a helical chromatid organization. The diagram shows the contact probabilities of different polymer models predicted for equilibrium and fractal globules, for simulated helical organization as suggested by Gibcus et al. (2018) and simulated half-helical arrangement with changing handedness as suggested by Chu et al. (2020). In all spatial models the polymer is coloured as shown for the unfolded polymer. For the helical and half-helical organizations, the polymer is transparent, and the basis of the loops appear as white spheres arranged in turns. Only the helical model creates the contact probability bump.

Table S1. Statistics of the metaphase Hi-C datasets. Seven libraries were analyzed: three replicates from interphase libraries, three replicates from metaphase libraries and one more metaphase library generated with an alternative protocol, which yields less unspecific contacts, for comparison with polymer modelling. For each library we computed: total number of contact reads (all reads), percentage of PCR duplicated reads (duplicated), number of non-duplicated reads with a mapping quality (MAPQ) of at least 10 (high-quality reads), number of intra-chromosome contacts separated by more than 1 kb (1 kb separation), intra-chromosome contacts separated by more than 1 Mb (1 Mb separation), and number of inter-chromosome contacts (inter-chromosome). * percentage of all reads: ** percentage relative to number high-quality reads; *** percentage of the reads separated by more than 1 kb.

LIBRARY	ALL READS	DUPLICATED (%*)	HIGH-QUALITY READS (%*)	1KB SEPARATION (%**)	1MB SEPARATION (%***)	INTER-CHROMOSOME (%***)
**INTERPHASE_1**	260,968,878	13.21	39,558,947 (15.16)	16,036,382 (40.54)	7,110,811 (44.34)	4,138,138 (25.8)
**INTERPHASE_2**	204,856,338	1.19	31,097,110 (15.18)	18,557,444 (59.68)	8,604,498 (46.37)	4,567,769 (24.61)
**INTERPHASE_3**	219,294,615	1.49	36,292,924 (16.55)	35,634,600 (98.19)	12,389,518 (34.77)	18,915,266 (53.08)
**METAPHASE_1**	509,196,403	6.06	91,401,051 (17.95)	62,357,229 (68.22)	12,764,657 (20.47)	48,635,089 (77.99)
**METAPHASE_2**	735,344,585	8.28	92,941,157 (12.64)	69,241,898 (74.5)	23,853,444 (34.45)	41,427,153 (59.83)
**METAPHASE_3**	579,559,917	7.09	80,301,755 (13.86)	60,884,103 (75.82)	17,350,884 (28.5)	40,922,614 (67.21)
**METAPHASE_AGAR**	122,636,722	50.74	12,194,950 (9.94)	9,809,014 (80.43)	4,771,893 (48.65)	3,254,155 (33.17)

Table S2. Values of local turn length of each chromosome calculated for 5 Mb bins, as described Figure S2. These values are graphically shown in Figs. 1E and S4. The three first lines display the sequence length of each chromosome, and the positions of the primary (centromere) and secondary constrictions (NORs) at the short arms of 5H and 6H, as predicted from Hi-C data. The oligo-FISH labelled region on chromosome 5HL is labelled in different colors according to the used bird probes.

	1H	2H	3H	4H	5H	6H	7H
Chromosome size (Mb)	522.5	675.3	628.7	624.4	599.0	573.2	634.7
Centromere position (Mb)	205.5	305.8	271.9	282.4	206.0	260.0	328.8
NORposition (Mb)	-	-	-	-	50.3	79.5	-
**Position (Mb)**	**Turn length (Mb)**						
**2.5-7.5**	6.01674	3.41191	10.0504	5.60E-08	5.96904	4.30E-08	5.47E-09
**7.5-12.5**	3.3989	3.39141	1.24E-07	2.38075	19.6863	6.14956	5.96E-07
**12.5-17.5**	13.6668	13.0618	11.0963	12.0694	22.1876	13.5131	6.32619
**17.5-22.5**	19.7629	17.18	18.8189	18.0119	24.3624	21.5796	15.2778
**22.5-27.5**	25.619	19.5063	23.289	22.9052	25.8212	25.1436	20.3829
**27.5-32.5**	26.3202	22.5116	26.0755	25.7573	26.2016	27.3088	22.4413
**32.5-37.5**	28.6499	24.3615	27.7775	27.5784	27.6527	29.2685	23.6694
**37.5-42.5**	29.4102	25.5515	29.5156	29.6878	26.864	30.6588	25.1945
**42.5-47.5**	30.4477	26.9644	30.6173	30.8019	13.6946	31.102	25.793
**47.5-52.5**	30.447	28.0789	31.1308	31.3201	3.90E-08	30.6038	25.6596
**52.5-57.5**	30.3814	28.9819	32.008	31.8613	1.22E-07	29.8517	25.8668
**57.5-62.5**	30.7721	29.178	32.1477	32.8573	25.2976	27.7301	25.6354
**62.5-67.5**	30.6446	29.6516	32.7131	33.4771	22.4426	27.0497	26.2038
**67.5-72.5**	30.8399	30.7569	33.2433	33.8482	26.4577	26.0902	26.9197
**72.5-77.5**	31.2262	31.1721	32.958	34.242	29.1869	25.5726	28.3646
**77.5-82.5**	32.7546	31.5567	33.5336	34.3294	30.456	1.99E-12	28.5584
**82.5-87.5**	33.2509	31.2866	33.3311	34.6787	30.8924	1.54E-07	29.7553
**87.5-92.5**	34.091	31.3926	33.6411	34.8001	32.0145	25.3131	30.1972
**92.5-97.5**	34.819	31.5472	34.1038	35.2321	32.6105	25.6364	30.681
**97.5-102.5**	34.9206	31.4086	33.9768	35.0999	32.5178	27.1352	30.9678
**102.5-107.5**	35.4487	31.6383	34.6804	34.9774	32.1115	28.9428	31.1725
**107.5-112.5**	35.2697	31.4884	34.6192	35.2558	31.4494	30.3431	31.8339
**112.5-117.5**	34.7301	31.8003	34.8457	35.4576	30.6382	31.6458	32.1062
**117.5-122.5**	34.4552	31.733	35.1639	35.6888	31.0143	32.8772	32.3051
**122.5-127.5**	33.6035	31.9941	35.1249	36.0441	30.4393	33.6562	32.4979
**127.5-132.5**	32.8825	32.1363	35.4935	36.2429	29.8152	34.4415	33.0943
**132.5-137.5**	32.0877	32.9942	35.6706	36.4408	29.8695	34.5958	33.4276
**137.5-142.5**	30.6648	33.1226	35.3801	36.8475	29.2471	34.7721	33.8254
**142.5-147.5**	30.5385	33.591	35.0965	37.2356	29.4943	34.8878	33.4321
**147.5-152.5**	29.2401	33.6246	34.9161	37.4198	31.0086	35.2648	32.5287
**152.5-157.5**	28.9405	33.6886	34.9754	37.4845	30.27	36.1125	36.2879
**157.5-162.5**	28.4541	33.7891	34.8313	37.2513	29.1436	36.69	37.0673
**162.5-167.5**	27.8497	33.3587	34.8597	37.298	28.0698	37.1208	36.0702
**167.5-172.5**	26.2151	33.4029	34.3964	37.2553	25.5765	37.2073	31.7659
**172.5-177.5**	23.036	34.0477	34.0597	36.7538	22.9311	37.364	33.1757
**177.5-182.5**	18.3452	34.2014	33.3938	35.8105	13.1703	37.2536	33.0345
**182.5-187.5**	3.23232	34.2238	32.9613	35.0615	8.88E-09	36.7446	32.3794
**187.5-192.5**	5.77E-11	34.066	32.8202	34.7704	3.68E-08	36.3706	35.1794
**192.5-197.5**	4.26E-08	34.0345	32.7427	33.9533	1.34E-07	35.8814	37.3129
**197.5-202.5**	9.58E-10	33.9191	32.4727	32.2715	6.15E-09	34.4791	37.6781
**202.5-207.5**	1.14E-08	34.0749	32.1912	31.6223	9.65E-08	33.876	33.5086
**207.5-212.5**	4.94E-09	33.8319	31.8995	33.0899	1.26E-07	32.5153	34.6433
**212.5-217.5**	4.44E-15	33.7478	31.137	32.4271	1.36E-08	30.1152	35.8472
**217.5-222.5**	2.10E-07	33.3611	29.9599	31.3009	4.68E-07	28.3225	36.1778
**222.5-227.5**	7.7988	32.7032	29.071	31.0981	4.35708	26.2381	36.2295
**227.5-232.5**	20.0756	32.9118	27.7213	32.0108	22.6128	23.3928	36.4219
**232.5-237.5**	24.9688	32.6444	26.2091	30.793	26.6324	14.8782	36.5365

	1H	2H	3H	4H	5H	6H	7H
Position (Mb)				Turn length (Mb)			
**237.5-242.5**	27.9133	32.9266	21.9091	30.7244	28.6671	2.99E-07	36.2472
**242.5-247.5**	30.6028	32.0568	18.0876	28.7014	30.7917	7.12E-10	36.0978
**247.5-252.2**	32.3382	32.0024	7.76712	25.1582	32.184	1.36E-08	36.3516
**252.5-257.5**	33.1497	31.8705	7.42E-08	22.4684	33.04	7.06E-09	36.0632
**257.5-262.5**	33.7869	30.3695	2.14E-09	12.6957	33.606	1.45E-09	35.4676
**262.5-267.5**	34.3179	29.3664	5.56E-07	4.58E-11	33.6956	4.81E-08	35.0643
**267.5-272.5**	34.2778	27.3048	2.19E-06	3.78E-10	31.9944	2.80618	34.5308
**272.5-277.5**	34.3777	24.7343	1.70E-08	5.06E-09	32.4848	1.60E-07	33.081
**277.5-282.5**	34.6652	19.9785	2.99E-08	3.56E-08	33.2126	8.64977	31.6357
**282.5-287.5**	34.6182	12.539	1.21E-09	8.74E-07	34.098	20.4693	30.8667
**287.5-292.5**	34.519	2.44E-13	6.19E-12	2.85E-06	34.7032	25.0188	28.3714
**292.5-297.5**	34.1255	1.19E-06	10.0299	6.00E-13	34.7975	27.3963	27.165
**297.5-302.5**	33.8657	9.33E-13	23.7339	14.3295	34.5394	28.6435	22.2139
**302.5-307.5**	33.0197	8.39E-10	26.1005	21.6783	34.7994	29.3851	17.3955
**307.5-312.5**	33.1925	6.59E-10	30.108	14.0763	34.4565	33.7904	2.33809
**312.5-317.5**	33.4299	1.79E-08	32.716	22.1995	34.907	34.8424	3.94E-07
**317.5-322.5**	33.4936	7.70E-10	34.2683	26.508	35.0075	33.2338	4.64E-07
**322.5-327.5**	33.2605	9.41484	35.4378	27.5564	34.6288	34.2273	1.83E-06
**327.5-332.5**	32.7137	19.3441	36.6982	28.5718	34.7095	34.1128	19.4349
**332.5-337.5**	32.6454	22.8902	37.4345	30.9083	34.6917	34.5335	5.71E-08
**337.5-342.5**	32.088	26.9874	37.5309	31.8386	34.3407	34.5175	1.43E-09
**342.5-347.5**	31.8489	27.8013	36.8835	32.6608	34.2121	34.2827	1.48E-10
**347.5-352.5**	31.4395	31.0436	36.1659	33.8463	33.9803	34.5684	6.32615
**352.5-357.5**	31.0148	31.8322	35.7429	32.9708	34.01	34.6865	18.7447
**357.5-362.5**	31.3222	33.8545	35.2958	33.1092	34.0151	34.7757	24.8626
**362.5-367.5**	31.0258	34.6337	34.8958	32.6703	33.9587	34.9235	26.6516
**367.5-372.5**	30.9657	34.973	34.3256	33.118	33.9184	34.394	28.9271
**372.5-377.5**	30.9108	35.6196	33.4739	32.3202	33.6151	34.6627	31.3187
**377.5-382.5**	30.6854	35.6359	32.4056	33.0433	33.2048	34.3154	32.6222
**382.5-387.5**	30.726	35.6518	32.246	32.7307	33.4495	33.848	34.0566
**387.5-392.5**	30.9995	35.5138	33.215	31.5889	33.2956	33.5464	34.4456
**392.5-397.5**	31.3994	35.1389	33.804	32.0617	33.2773	33.8538	34.8513
**397.5-402.5**	31.0889	34.4437	34.0328	33.5815	33.1836	33.8478	35.2768
**402.5-407.5**	30.4093	33.7706	33.9275	34.0165	32.954	33.9513	35.0858
**407.5-412.5**	29.8711	33.7733	34.2662	34.1398	32.5537	33.8335	34.8808
**412.5-417.5**	29.1366	32.7707	34.1763	34.3838	32.1396	33.6421	34.9159
**417.5-422.5**	29.1749	32.1647	34.0588	34.8112	32.1213	34.6381	34.4724
**422.5-427.5**	28.6555	32.9012	33.7376	34.6444	31.8305	33.6526	34.0792
**427.5-432.5**	28.9366	32.9395	33.8082	35.519	31.2156	34.2223	34.1023
**432.5-437.5**	28.2459	33.4327	33.6258	34.8364	31.0672	34.1226	33.8582
**437.5-442.5**	28.9684	33.7228	33.1357	34.2773	30.6198	33.7286	33.8694
**442.5-447.5**	28.2041	33.89	32.7506	33.8143	30.3953	32.994	34.0414
**447.5-452.5**	28.2515	34.0404	32.2745	33.3632	30.0231	33.0358	33.8713
**452.5-457.5**	27.5916	34.4113	32.2374	33.5331	29.8501	32.2564	33.3055
**457.5-462.5**	26.291	34.2249	32.136	32.7541	29.6158	31.5048	33.2865
**462.5-467.5**	25.9303	34.5612	31.6487	32.743	29.184	32.3898	33.4259
**467.5-472.5**	25.4411	34.3868	31.4897	33.4705	29.0229	31.822	33.2463
**472.5-477.5**	23.9437	34.1271	31.4121	32.9942	29.1223	31.0097	33.3311
**477.5-482.5**	23.3546	34.209	30.8674	33.3189	27.7607	31.2104	33.4033
**482.5-487.5**	22.6197	34.0958	30.4565	33.4761	26.0865	31.0838	33.3216
**487.5-492.5**	21.7581	34.1659	29.9014	33.1369	25.7346	31.4538	33.0992
**492.5-497.5**	20.3533	34.0828	29.4672	33.0742	24.8074	30.3505	33.0381
**497.5-502.5**	17.3684	34.4562	28.3903	32.7133	23.9356	29.4287	32.731
**502.5-507.5**	12.2531	33.7769	28.2051	32.3605	22.5968	29.4079	32.3883
**507.5-512.5**	3.78151	33.7908	26.9858	32.0161	22.4535	29.1924	31.7152
**512.5-517.5**	12.0529	33.1738	26.6734	31.5706	20.9526	28.1931	32.112
**517.5-522.5**	6.13769	32.9463	26.9733	30.9844	21.5121	27.3251	32.3378
**522.5-527.5**		32.4902	26.0259	30.59	21.265	27.2698	31.6343
**527.5-532.5**		31.8125	26.2868	30.093	21.0415	26.2253	31.6827

	1H	2H	3H	4H	5H	6H	7H
**Position (Mb)**				Turn length (Mb)			
**532.5-537.5**		31.5727	26.074	29.7905	20.4688	25.9744	31.4727
**537.5-542.5**		30.5718	25.5686	30.3023	21.187	25.6962	31.7087
**542.5-547.5**		29.8406	25.6092	29.9643	21.6532	23.5544	31.4402
**547.5-552.5**		29.2722	25.4209	29.2289	22.4012	21.8487	30.7147
**552.5-557.5**		29.0004	24.5428	28.6189	24.049	17.465	30.7752
**557.5-562.5**		27.9736	23.806	28.1934	23.1059	11.6061	30.1034
**562.5-567.5**		27.0287	24.2538	27.9885	23.353	6.49198	29.6873
**567.5-572.5**		27.4258	23.9249	27.339	21.6832	3.90E-07	29.1887
**572.5-577.5**		26.6489	23.1854	26.4052	19.8346		28.5508
**577.5-582.5**		25.0741	24.0151	25.3589	17.2077		28.2294
**582.5-587.5**		23.595	23.3074	24.1774	7.7922		26.6481
**587.5-592.5**		23.6982	22.2953	23.2408	8.17889		26.2341
**592.5-597.5**		24.8433	20.5223	23.2727	1.51E-06		24.7005
**597.5-602.5**		24.6947	20.2942	20.5142			23.5091
**602.5-607.5**		24.7661	18.5698	17.4219			21.548
**607.5-612.5**		23.5646	16.2229	11.4805			19.0632
**612.5-617.5**		22.9534	13.6966	5.22E-09			15.5532
**617.5-622.5**		22.9203	14.5255	3.97E-08			5.2426
**622.5-627.5**		21.6953	10.2715				1.24672
**627.5-632.5**		21.5698					1.01E-11
**632.5-637.5**		22.3165					
**637.5-642.5**		22.1064					
**642.5-647.5**		21.342					
**647.5-652.5**		20.4072					
**652.5-657.5**		17.5867					
**657.5-662.5**		10.5045					
**662.5-667.5**		4.48304					
**667.5-672.5**		7.678					
**672.5-677.5**		13.348					

Table S3. Coordinates and lenghts of the oligo-FISH probes (named according to birds) adjacent to the 5HL subtelomere based on the barley cv. Morex v2 genome assembly.

Probe	No. oligos	Start (Mb)	End (Mb)	Length (Mb)	Average Turn size (Mb)	Number of turns*
**Stork**	33987	442.1	459.9	17.7	30.1±0.4	0.6
**Eagle**	35062	459.9	477.5	17.6	29.2±0.2	0.6
**Ostrich**	91061	477.4	511.8	34.4	24.8±1.8	1.4
**Rhea**	105984	511.8	545.3	33.5	21.3±0.5	1.58
**Moa**	108831	545.3	580.2	34.9	21.6±2.1	1.62
**Flamingo**	62181	580.1	599.0	18.8	8.3±6.1	2.3

* Length of the probe divided by the average turn size in its region.

Table S4. Volumes of the barley satellite chromosomes 5H and 6H (identified based on their secondary constriction and centromere position) and NOR-free (1H-4H, 7H) chromosomes. The measurements were done on polyacrylamide embedded spatially preserved chromosomes.

Chromosome	n	Mean±SD (μm³)
**5H**	2	49.5±2.3
**6H**	3	55.0±12.8
**1H-4H, 7H**	10	56.1±9.8
**1H-7H**	15	55.0±10.1

Supplementary movies

All the movies are available in the link: http://dx.doi.org/10.5447/ipk/2023/2

Hi-C sliding window analysis

Movie S1 (Hi-C_sliding_window_analysis.mp4): Bump position along chromosome 5H indicating the turn sizes. Placement of the bump calculated for 119 different positions in the sequence of chromosome 5H separated by 5 Mb.

FISH

Movie S2 (FISH_1.avi). The 14 somatic barley metaphase chromosomes labelled by FISH as described in Figures. 2A, B, S6. The movie shows clearly that the oligo-FISH labelled 5HL region is also labelled inside of the chromosome arms.

Movie S3 (FISH_2.mp4). The slices of a chromosome 5H 3D-SIM image stack shown in ortho view (Fig. 2C) indicate the complete FISH labelling of the inner chromatin.

Movie S4 (FISH_3.avi). Spatial minor chromatin fiber intermingling of adjacent chromonema turns at chromosome arm 5HL labelled in different colors by oligo-FISH (Fig. S10).

Movie S5 (FISH_4.mp4). The spatial surface rendering of chromosome 5H (Fig. 2D) displays that the FISH signals are present within the whole chromosome volume.

Movie S6 (FISH_5.mp4). Spatial arrangements of telomeres and subtelomeres (Fig. 2I).

Chromatin structure - no cavities

Movie S7 (Chromatin_structure-no_cavities_1.avi). The single SIM image stack slices of a DAPI-labelled 6H chromosome show a network of minor ~80 nm looped chromatin fibers free of large cavities throughout the chromosome arms. Within centromere and NOR straight ~80 nm chromatin fibers occur.

Movie S8 (Chromatin_structure-no_cavities_2.mp4). Spatial organization of the same 6H chromosome shown in Movie S7.

SCEs

Movie S9 (SCEs_1.mp4). Spatial arrangement of the oligo-FISH and EdU labelled region of the left 5H chromosome (*) shown in (Fig. 4).

Movie S10 (SCEs_2.mp4). Spatial arrangement of the oligo-FISH and EdU labelled region of the right 5H chromosome (**) shown in (Fig. 4).

Polymer simulation model

Movie S11 (Polymer_simulation_model.mp4). Polymer simulation model shown in (Fig. 1G) for a single FISH labelled 5H chromatid.
